# Research on hybrid estimation algorithm for dynamic center-of-mass parameters of tracked agricultural transport platform

**DOI:** 10.3389/fpls.2026.1928457

**Published:** 2026-10-13

**Authors:** Yitian Wang, Daohong Chong, Jinxin Luo, Longkai Liang, Yangyang Zou

**Affiliations:** 1 College of Mechanical and Electrical Engineering, Changchun University of Science and Technology, Changchun, Jilin, China; 2 College of Instrumentation and Electrical Engineering, Jilin University, Changchun, Jilin, China; 3 College of Artificial Intelligence, Changchun University of Science and Technology, Changchun, Jilin, China

**Keywords:** agricultural transport platform, deep learning, hybrid estimation algorithm, smart agriculture, unstructured environment

## Abstract

To address dynamic centroid shift and reduced control accuracy caused by complex terrain and multi-device collaborative loading on tracked agricultural transport platforms, a hybrid estimation algorithm (CBA-IPSO-UKF) is proposed. This algorithm is designed for platforms operating in unstructured outdoor environments and combines a deep learning-based model with intelligent optimization techniques. First, the limitations of traditional UKF algorithms—which depend heavily on physical models and struggle with strong nonlinear disturbances—are overcome by developing a CNN-BiLSTM hybrid model with attention mechanisms. This model replaces conventional state and observation equations. Second, to reduce systematic errors from model mismatch, a constant-coefficient compensation vector is incorporated into the estimation framework. An improved particle swarm optimization algorithm, enhanced with genetic algorithm crossover and mutation operators, is used to optimize global parameters and minimize residuals. Real-vehicle data were collected from a self-developed tracked agricultural platform, resulting in a dataset of 18, 000 valid samples. Comparative experiments were carried out under typical circular driving conditions in unstructured environments. The results demonstrate that the proposed algorithm achieves high accuracy and steady-state performance in both attitude and velocity estimation. Specifically, the mean absolute errors for roll angle, pitch angle, and heading angle were 0.0917°, 0.1352°, and 3.1458°, respectively. The MAE for the three-axis velocities reached 0.0622 m/s, 0.0616 m/s, and 0.0209 m/s, respectively. These results show significant improvements over traditional algorithms like UKF and ACKF. This study offers both theoretical support and practical engineering solutions for real-time state perception and the stability control of autonomous operations in complex environments for intelligent agricultural machinery.

## 1 Introduction

With the rapid advancement of smart agriculture and autonomous farming, stricter requirements have been imposed on the self-operating capabilities of tracked agricultural transport platforms. By utilizing their excellent terrain adaptability, these platforms are crucial for transporting materials in unstructured farmland environments (Yu et al., 2025a; Liu et al., 2026). However, multi-equipment collaborative loading on complex terrains—such as terraced slopes (as shown in **Figure 1**)—easily causes dynamic shifts in the center of mass (CoM). This results in strong nonlinear system behaviors that significantly limit attitude control accuracy and driving stability. At the same time, limitations in onboard sensor precision and existing estimation algorithms hinder real-time, accurate measurement of dynamic CoM parameters (Wang et al., 2024; Koide et al., 2025; Gao et al., 2023). Therefore, developing a parameter estimation framework focused on dynamic CoM under nonlinear, complex operating conditions, to enable real-time, precise perception of platform states, has become essential. Overcoming control challenges in intelligent agricultural transport systems and ensuring safe autonomous operations are key to achieving both significant theoretical and practical value.

**Figure 1:**
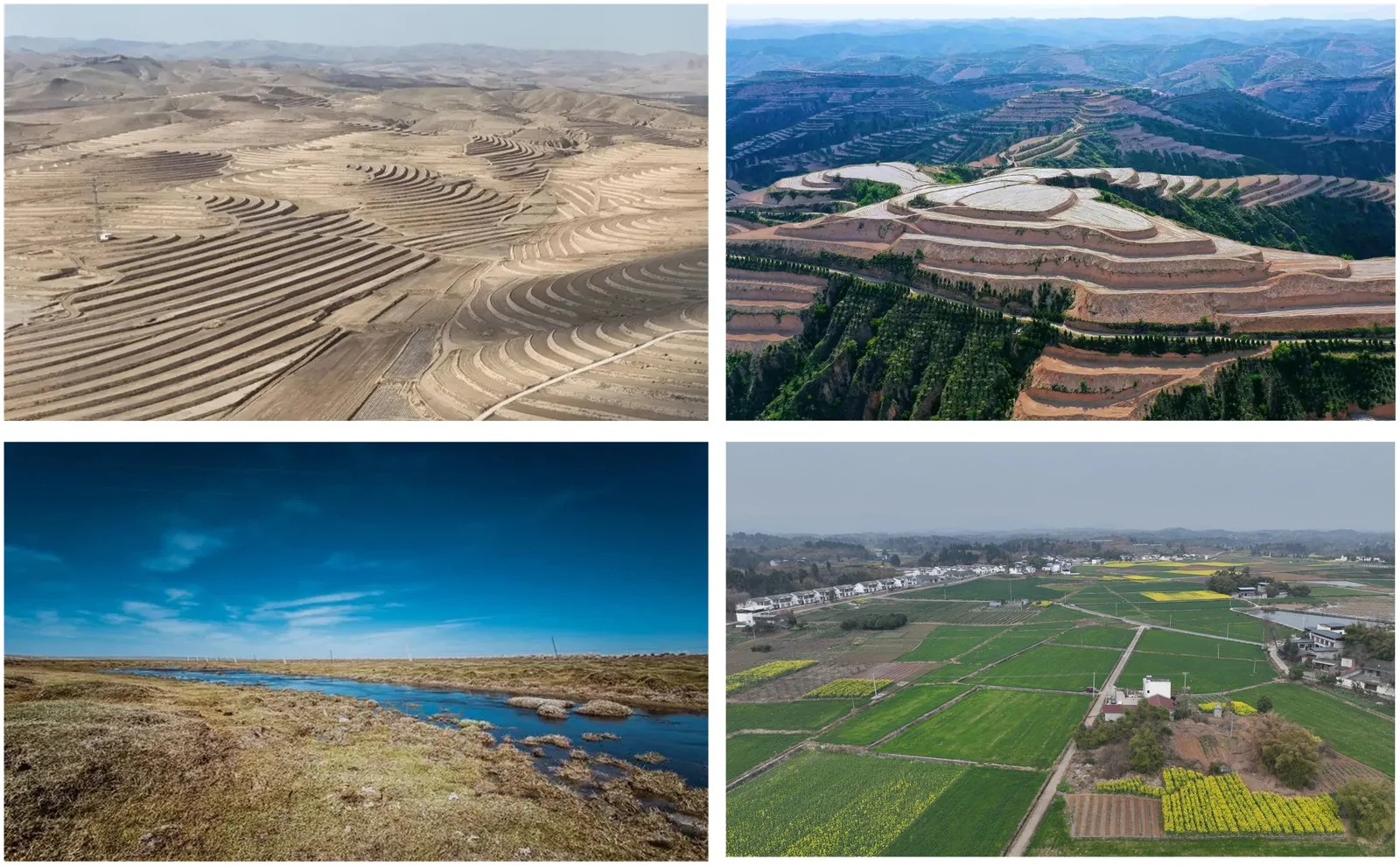
Overview of common unstructured field environments and their terrain features.

Dynamic parameter estimation of the center of mass is crucial for achieving high-precision autonomous operations in agricultural equipment. This subject has garnered significant academic interest over the years (Habibnejad Korayem et al., 2022). Current estimation algorithms primarily utilize two technical approaches. One relies on kinematic and dynamic modeling methods based on physical prior knowledge. The other depends on data-driven techniques that utilize onboard sensor data.

In research based on kinematic and dynamic mechanism models, Li et al. developed a longitudinal dynamic coupling model for electric tractors tailored to the load characteristics of tillage and transportation operations. They derived state differential equations for the longitudinal and angular acceleration of the center of mass and demonstrated the system’s observability (Li et al., 2024). Wang et al. developed a seven-DOF vehicle dynamics model with the Dugoff tire model and used UKF to estimate the center-of-mass yaw angle for four-wheel-drive electric vehicles (Wang et al., 2023). Tufano et al. proposed an estimation algorithm based on an Interactive Multi-Model filter, utilizing a two-degree-of-freedom monorail model for real-time observation of the center-of-mass yaw angle. Its robustness under sudden changes in road surface friction coefficients was validated through full-vehicle experiments (Tufano et al., 2024). Additionally, Wang et al. employed a high-fidelity nine-degree-of-freedom nonlinear model. They addressed modeling errors caused by dynamic changes in the center of mass position through multi-parameter decoupling estimation (Wang et al., 2023). However, these model-based approaches heavily depend on precise physical parameters. Given the complex operating environments and significant model uncertainties in agricultural machinery, such methods are prone to decreased estimation accuracy or divergence.

Besides mechanism modeling, direct observation using onboard sensors is also crucial. Its ability to provide absolute spatial references and ease of deployment has made it an essential part of intelligent agricultural machinery perception (Huang et al., 2025). Among existing studies, Zhang et al. proposed a deep learning approach to identify turning patterns in GPS trajectories of agricultural machinery, enhancing the accuracy of operational efficiency evaluations in complex farmland environments (Zhang and Fang, 2024). Azmi et al. analyzed tractor trajectories in oil palm plantations using built-in GPS and quantitatively assessed the straight-line tracking performance of an automatic navigation system (Azmi et al., 2022). Chou et al. integrated GPS into the steering control system of an all-terrain vehicle and verified its safety control effectiveness under normal operating conditions (Chou et al., 2022). However, during field boundary or in-forest operations, satellite signals are vulnerable to occlusion by buildings, vegetation, and terrain, leading to loss of lock or multipath interference. This causes reduced positioning accuracy or even signal loss, compromising the reliability of motion control (Zhou et al., 2020).

Given GNSS limitations, the inertial measurement unit (IMU) acts as a self-sensing sensor that does not depend on external reference signals. It allows continuous attitude and position estimation in environments without satellite signals, making it particularly useful for short-term, accurate measurements in complex agricultural operations (Jiang et al., 2022). Zhao et al. proposed a multi-sensor fusion approach combining IMU, encoder, and LiDAR data to develop a joint estimation framework for steering angle, yaw moment of inertia, and tire lateral stiffness (Zhao et al., 2021). To address installation errors between IMUs and onboard sensors, Xia et al. developed an autonomous yaw bias estimation algorithm validated through real-vehicle testing (Xia et al., 2022). Yan et al. introduced a deep fusion architecture that merges visual-inertial odometry with wheel odometry to improve the state perception capabilities of agricultural robots in dynamic environments (Yan et al., 2022). However, IMUs are vulnerable to mechanical vibrations and random noise interference in unstructured, rugged terrain. Additionally, the zero-bias instability inherent in inertial components causes error accumulation over time, leading to state estimation divergence and making it challenging to meet accuracy requirements for long-cycle operations (Patrizi et al., 2023; Hong et al., 2024; Li et al., 2024).

Based on existing research, as the dynamic complexity of controlled systems increases, Kalman filtering and its derivative algorithms, which are based on statistical principles, have gained significant attention. These methods are valued for their excellent noise reduction capabilities (Geng et al., 2025; Ghanizadegan and Hashim, 2025; Caglio et al., 2025). Hoang et al. proposed a tightly coupled lidar-vision-inertial odometry scheme for autonomous agricultural robots. They used an error state Kalman filter for pose estimation to effectively reduce measurement noise and system uncertainty in unstructured environments (Hoang and Kim, 2024). Lv et al. developed an extended Kalman filter-based multi-sensor fusion framework to address environmental interference in agricultural robot navigation. By loosely combining IMU, odometer, GPS, and visual-inertial odometry data, they significantly increased positioning accuracy under sensor-failure conditions (Lv et al., 2022). Jensen et al. proposed a method for estimating passenger vehicle mass that used only IMU and GNSS data, without CAN bus input. Using a Kalman filter fusion algorithm to account for slope and inertial forces, they markedly improved the accuracy of vehicle mass estimation (Jensen et al., 2025). Seo et al. introduced a model-free velocity estimator based on the Extended Kalman Filter. They built motion equations using the RVST relationship and an observation model that combines IMU and wheel-speed data. Their approach demonstrated better estimation performance under complex conditions compared to traditional model-based methods (Seo and Kang, 2025).

However, traditional estimation algorithms still struggle to handle highly nonlinear errors and adapt to complex environments. To address this, researchers have introduced artificial intelligence algorithms with strong adaptive capabilities to assist with state prediction (Chinellato and Marcuzzi, 2025; Xu et al., 2024). Gao et al. proposed the Gyro-Net learning method to estimate and compensate for gyro random errors online, thereby increasing IMU attitude estimation accuracy (Gao et al., 2023). Guo et al. proposed a hybrid framework for accurate error parameter identification in hypersonic vehicle inertial navigation systems (Guo et al., 2020). Sharifi et al. proposed the RTA-NN method, which deeply integrates neural networks with Kalman filters to achieve high-precision transfer alignment between master and slave devices (Sharifi et al., 2025). To address the nonlinear and non-stationary characteristics of state estimation under complex conditions, multi-source algorithm fusion has become a key approach to boosting performance. Ge et al. built on the Square Root Volume Kalman Filter by optimizing the Broadly Learned System’s regression parameters using particle swarm optimization, effectively improving the overall accuracy of the Kalman filter (Ge et al., 2025). Wang et al. developed a joint estimation framework that combines an enhanced particle swarm optimization algorithm with the Kalman particle filter. By incorporating an improved recursive least-squares method with a forgetting factor and a memory length, they achieved high-precision, online identification of time-varying system parameters (Wang et al., 2025). Xing et al. applied an improved cuckoo search algorithm to optimize the volume Kalman filter, enhancing its convergence capabilities in complex nonlinear environments. Validation using real ocean datasets demonstrated that this method effectively improved the reliability of trajectory tracking for underwater vehicles (Xing et al., 2026).

Nevertheless, accurately estimating the dynamic parameters of the center of mass in agricultural equipment remains a significant challenge. During field operations, environmental disturbances and dynamic loads cause the distribution of the center of mass to vary greatly over time. Traditional mechanism-based modeling methods are limited by the strong nonlinearity of vehicle structures, making it difficult to balance estimation accuracy and real-time performance. This paper proposes a deep learning-based architecture for dynamic center-of-gravity estimation. By combining the nonlinear mapping abilities of deep neural networks with the search advantages of intelligent optimization algorithms, it avoids the complexities of physical modeling, achieving robust observation and high-precision estimation of the center-of-gravity state under challenging operating conditions.

In summary, this study presents a hybrid estimation algorithm for the dynamic center-of-mass parameters of tracked agricultural transport vehicles. The algorithm integrates deep neural networks with intelligent optimization techniques. It aims to achieve accurate parameter estimation under complex operating conditions. The research involves three steps. First, a CNN-BiLSTM-Attention hybrid model is developed to enhance the extraction of key dynamic features. Second, deep learning outputs are combined with an improved particle swarm optimization algorithm. This hybrid approach collaboratively refines the Unscented Kalman Filter for better convergence accuracy and stability in nonlinear state estimation. Lastly, the effectiveness of the proposed algorithm is validated via real-vehicle ground experiments.

## 2 Modeling and error calibration of IMU system

This study uses a nine-axis Inertial Measurement Unit (IMU) to capture three-dimensional motion features of the tracked agricultural transport platform. The unit combines tri-axial accelerometers *a_b_*=[*a_x_ a_y_ a_z_*], tri-axial gyroscopes *w_b_*=[*w_x_ w_y_ w_z_*], and tri-axial magnetometers *m_b_*=[*m_x_ m_y_ m_z_*]. Due to the physical constraints of the sensors, IMU outputs include both deterministic and stochastic errors, with deterministic errors primarily comprising systematic biases, such as scale factors, biases, and non-orthogonal mounting misalignments. To improve data quality, deterministic errors are calibrated offline using the IMU-TK toolbox, with calibration models shown in Equations 1–3:

(1) $$S_{a}=[\begin{array}{ccc} 1 & -0.02324 & 0.09464\\ 0 & 1 & 0.01911\\ 0 & 0 & 1 \end{array}]\;S_{g}=[\begin{array}{ccc} 1 & -0.01215 & -0.009\\ 0.01544 & 1 & -0.01108\\ -0.09213 & 0.0182 & 1 \end{array}]$$

(2) $$K_{a}=[\begin{array}{ccc} 0.9763 & 0 & 0\\ 0 & 0.93647 & 0\\ 0 & 0 & 0.9150 \end{array}]\;K_{g}=[\begin{array}{ccc} 0.9546 & 0 & 0\\ 0 & 0.9152 & 0\\ 0 & 0 & 0.9787 \end{array}]$$

(3) $$b_{a}=[\begin{array}{c} 0.964414\\ 0.139414\\ -0.906982 \end{array}]\;b_{g}=[\begin{array}{c} -0.00228\\ 0.005967\\ 7.982e-5 \end{array}]$$

In the formulas mentioned above, *S_a_* and *S_g_* represent scale factors, *K_a_* and *K_g_* represent installation non-orthogonality errors, and *b_a_* and *b_g_* represent zero offsets. After applying compensation using Equations 1–3, the actual angular velocity and acceleration can be calculated, as shown in Equations 4, 5:

(4) $$\left[\begin{array}{l} a_{x}\\ a_{y}\\ a_{z} \end{array}]=[\begin{array}{ccc} K_{axx} & K_{axy} & K_{axz}\\ 0 & K_{ayy} & K_{ayz}\\ 0 & 0 & K_{azz} \end{array}]\cdot [\begin{array}{ccc} S_{ax} & 0 & 0\\ 0 & S_{ay} & 0\\ 0 & 0 & S_{az} \end{array}]\cdot [\begin{array}{l} a_{x,true}\\ a_{y,true}\\ a_{z,true\;} \end{array}]-[\begin{array}{l} b_{ax}\\ b_{ay}\\ b_{az} \end{array}\right]$$

(5) $$\left[\begin{array}{l} g_{x}\\ g_{y}\\ g_{z} \end{array}]=[\begin{array}{ccc} K_{gxx} & K_{gyy} & K_{gzz}\\ 0 & K_{gy} & K_{gzz}\\ 0 & 0 & K_{gz} \end{array}]\cdot [\begin{array}{ccc} S_{gx} & 0 & 0\\ 0 & S_{gy} & 0\\ 0 & 0 & S_{gz} \end{array}]\cdot [\begin{array}{l} g_{x,true}\\ g_{y,true}\\ g_{z,true\;} \end{array}]-[\begin{array}{l} b_{gx}\\ b_{gy}\\ b_{gz} \end{array}\right]$$

The IMU uses a *Z*-*Y*-*X* rotation sequence (clockwise as positive). The rotation matrix that transforms the navigation coordinate system to the carrier coordinate system is shown in (Equation 6):

(6) $$C_{b}^{n}=[\begin{array}{ccc} \cos y\cos p & -\sin y\cos r+\cos y\sin p\sin r & \sin y\sin y+\cos y\sin p\cos r\\ \sin y\cos p & \cos y\cos r+\sin y\sin p\sin r & -\sin r\cos y+\sin y\sin p\cos r\\ -\sin p & \cos p\sin r & \cos p\cos r \end{array}]$$

In (Equation 6), *y*, *p*, and *r* represent the yaw, pitch, and roll angles of the platform’s center of mass, respectively.

The normalized gravitational acceleration in the navigation coordinate system is *a_n_*= [0, 0, –1], and its transformation to the body coordinate system is given by Equation 7:

(7) $$A_{b}=[\begin{array}{c} -\sin p\\ \cos p\sin r\\ \cos p\cos r \end{array}]=[\begin{array}{l} a_{x}\\ a_{y}\\ a_{z} \end{array}]$$

The pitch and roll angles of the centroid can be calculated from Equations 8, 9:

(8) $$p=\text{arcsin}(-a_{x})$$

(9) $$r=\arctan(a_{y}/a_{z})$$

Assume the navigation coordinate system is aligned with the body coordinate system, with the azimuth angle at 0 when the magnetometer’s *x*-axis points east. Under these conditions, the magnetometer’s output in the navigation coordinate system is described by Equation 10:

(10) $$m_{N}=[\begin{array}{lll} 0 & m_{n} & m_{u} \end{array}]$$

The conversion of magnetic field intensity from the navigation frame to the body frame is represented as:

(11) $$\left[\begin{array}{l} m_{x}\\ m_{y}\\ m_{z} \end{array}]=[\begin{array}{c} \sin y\cos p\times m_{n}-\sin p\times m_{u}\\ (\cos y\cos r+\sin y\sin p\sin r)\times m_{n}+\cos p\sin r\times m_{u}\\ (-\sin r\cos y+\sin y\sin p\cos r)\times m_{n}+\cos p\cos r\times m_{u} \end{array}\right]$$

(12) $$\left\{ \begin{array}{c} m_{x}=\sin y\cos p\times m_{n}-\sin p\times m_{u}\\ m_{y}=(\cos y\cos r+\sin y\sin p\sin r)\times m_{n}+\cos p\sin r\times m_{u}\\ m_{z}=(-\sin r\cos y+\sin y\sin p\cos r)\times m_{n}+\cos p\cos r\times m_{u} \end{array}\right.$$

From Equations 11, 12, the heading angle of the transport platform’s center of mass can be derived as Equations 13–15:

(13) $$y=\arctan(\frac{m_{x}\cos p+m_{y}\sin p\sin r+m_{z}\sin p\cos r}{m_{y}\cos r-m_{z}\sin r})$$

(14) $$v_{k}=[\begin{array}{l} v_{e_{k}}\\ v_{n_{k}}\\ v_{u_{k}} \end{array}]=[\begin{array}{c} v_{e_{k-1}}+\int_{k-1}^{k}\left(C_{b}^{n}a_{x}\right)\text{d}t\\ v_{n_{k-1}}+\int_{k-1}^{k}\left(C_{b}^{n}a_{y}\right)\text{d}t\\ v_{u_{k-1}}+\int_{k-1}^{k}\left(C_{b}^{n}a_{z}-g\right)\text{d}t \end{array}]$$

(15) $$x_{k}=[\begin{array}{l} x_{e_{k}}\\ x_{n_{k}}\\ x_{u_{k}} \end{array}]=[\begin{array}{c} x_{e_{k-1}}+v_{e_{k-1}}t+\iint_{k-1}^{k}(C_{b}^{n}a_{x})\text{d}t\\ x_{n_{k-1}}+v_{n_{k-1}}t+\iint_{k-1}^{k}(C_{b}^{n}a_{y})\text{d}t\\ x_{u_{k-1}}+v_{u_{k-1}}t+\iint_{k-1}^{k}(C_{b}^{n}a_{z}-g)\text{d}t \end{array}]$$

## 3 Proposed method

This section introduces a hybrid algorithm for real-time estimation of the center-of-mass parameters of tracked agricultural transport platforms, as shown in **Figure 2**. The core framework is based on the UKF, which incorporates a CNN-BiLSTM hybrid model with attention mechanisms to replace traditional state and observation models. Additionally, a compensation vector is introduced during state estimation, and an improved particle swarm optimization algorithm is employed to perform global optimization and reduce the residuals between estimated and actual values. Ultimately, the platform’s attitude and velocity states are updated in real time using this compensation mechanism.

**Figure 2:**
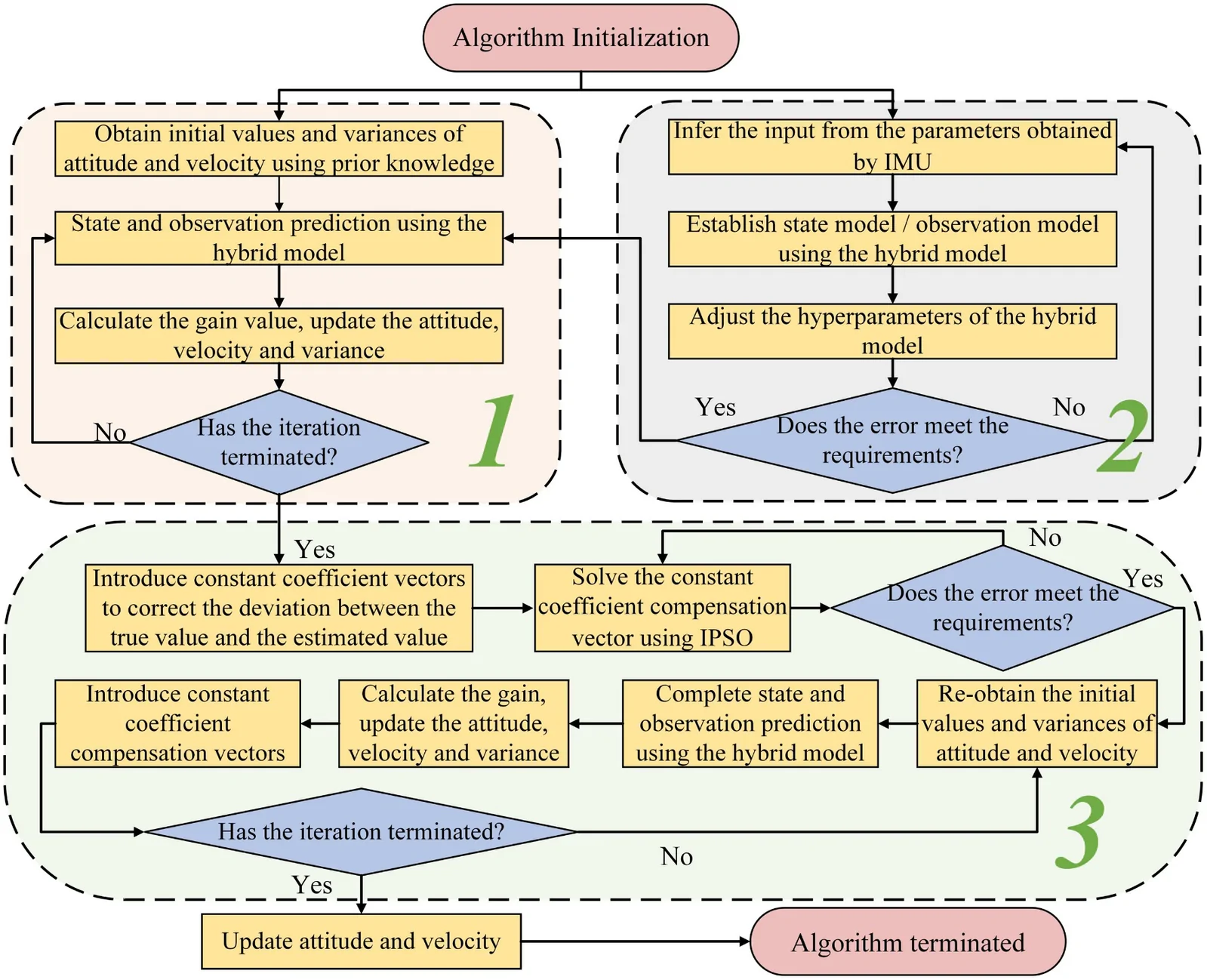
Overall workflow framework of the hybrid estimation method.

### 3.1 UKF algorithm

The Unscented Kalman Filter (UKF) handles state estimation in nonlinear systems using unscented transformations (Lee et al., 2021) and it has been widely applied in the estimation of agricultural machinery movement (Yu et al., 2025b, Xie et al., 2024). Its main idea is to linearize nonlinear functions with a deterministic set of sigma points to ensure high-order accuracy in state transfer. **Figure 3** shows the UKF iterative estimation structure.

**Figure 3:**
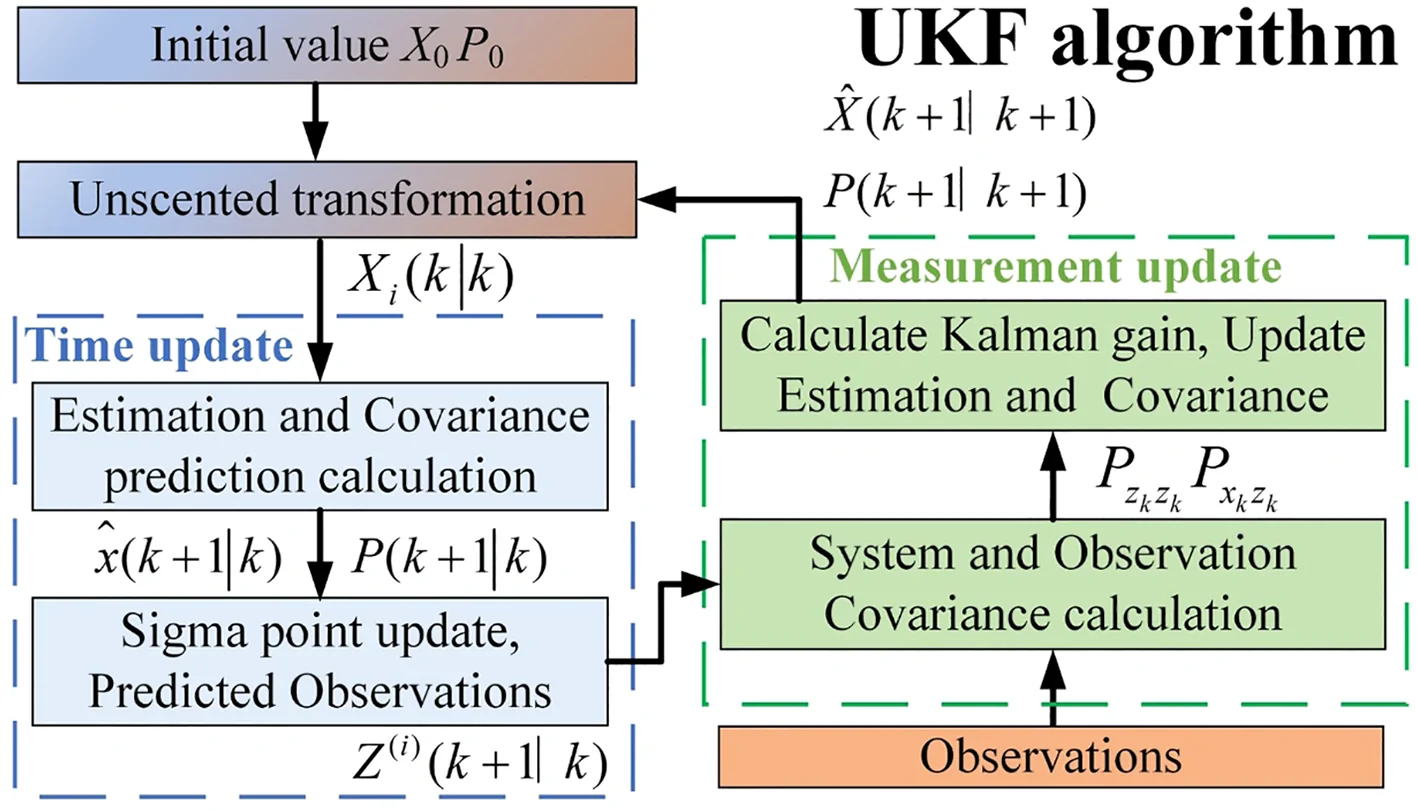
Iterative estimation architecture of the UKF algorithm.

Despite the promising outlook of UKF in state estimation, three main limitations remain: first, it depends heavily on models, requiring high accuracy in systems with unknown noise environments; second, the “curse of dimensionality, “ where the computational burden of sampling rises sharply during high-dimensional state estimation; and finally, the lack of effective adaptive compensation mechanisms, which makes it hard to fully eliminate systematic estimation errors caused by model mismatches at the algorithmic level. In response, this study proposes strategies to address these issues.

### 3.2 CNN-BiLSTM-attention hybrid model

To address the decline in estimation accuracy caused by highly nonlinear conditions in UKF, this study proposes a hybrid CNN-BiLSTM-Attention model as an alternative to traditional mathematical modeling methods. By thoroughly integrating CNN, BiLSTM, and attention mechanisms, this model achieves an end-to-end mapping between state prediction and observation update.

During the feature processing stage, the CNN module uses one-dimensional convolutional layers to extract local nonlinear features from state and observation data thoroughly, leveraging weight sharing to reduce computational complexity. Next, pooling layers decrease the spatial dimensions while preserving important features, thus enhancing feature robustness. The resulting feature vectors are concatenated and fed into the BiLSTM module, which leverages its bidirectional temporal modeling to analyze complex dynamic patterns further. This diverse architecture effectively overcomes the limitations of traditional analytical models, improving stability in state estimation under complex operating conditions (**Figure 4**).

**Figure 4:**
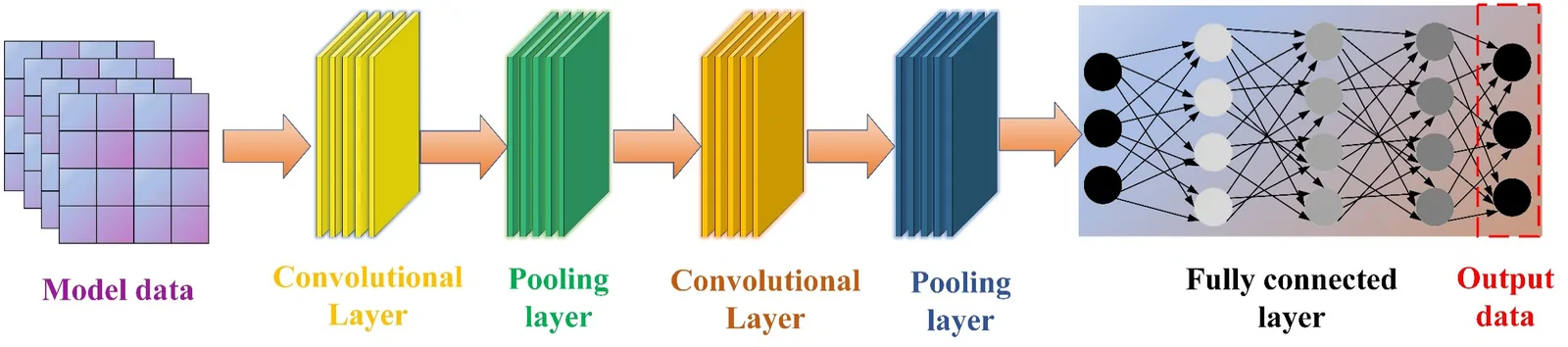
Convolutional neural network architecture.

As shown in **Figure 5a**, LSTM maintains long-term information flow through memory cells and gating mechanisms. Since unidirectional LSTM cannot utilize backward sequence features, this study introduces a bidirectional parallel-structured BiLSTM module (**Figure 5b**). This module thoroughly explores bidirectional dependencies in temporal data by combining forward and backward hidden layer states. Compared to unidirectional LSTMs, BiLSTMs provide a more comprehensive description of the dynamic evolution of state variables, enhancing feature learning accuracy under complex operating conditions (Dong et al., 2024).

**Figure 5:**
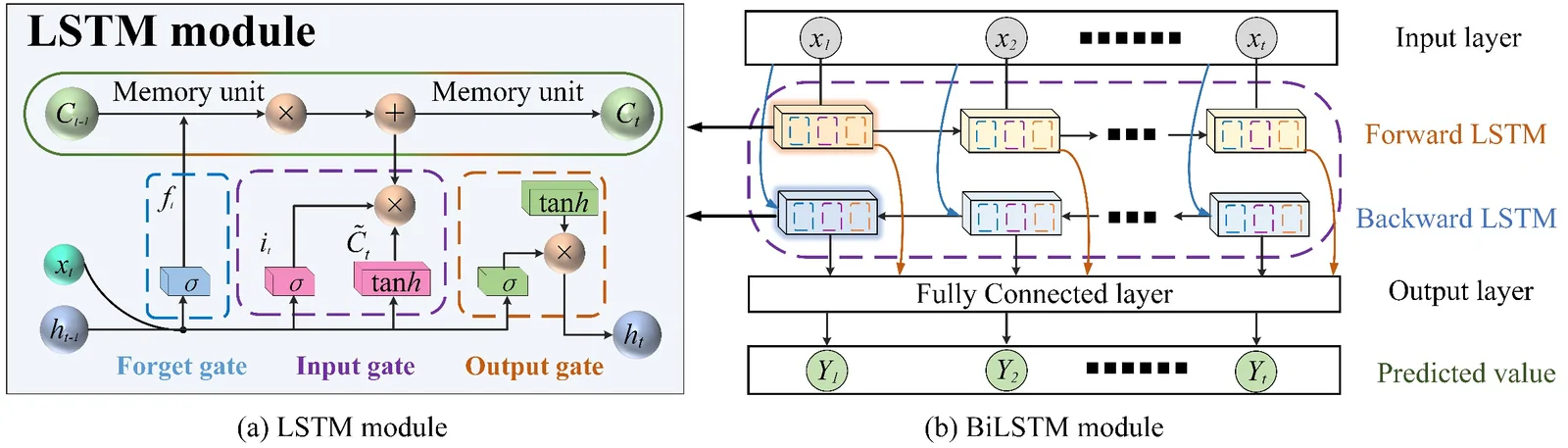
Network architecture comparison between LSTM and BiLSTM. **(a)** LSTM module. **(b)** BiLSTM module.

The structure of the BiLSTM module is illustrated in **Figure 5b**, which achieves bidirectional information fusion through the parallel configuration of forward and backward LSTM layers. Let *x_t_*denote the input features of this module at time *t*, $\vec{h_{t}}$ represent the hidden state output of the forward LSTM layer, and $\overset{\leftarrow }{h_{t}}$ represent the hidden state output of the backward LSTM layer. The final output features *Y_t_*at time *t* can then be obtained by concatenating $\vec{h_{t}}$ and $\overset{\leftarrow }{h_{t}}$. The complete update process of BiLSTM is presented in Equation 16.

(16) $$\left\{ \begin{array}{l} \vec{h_{t}}=LSTM(x_{t},\vec{h_{t-1}})\\ \overset{\leftarrow }{h_{t}}=LSTM(x_{t},\overset{\leftarrow }{h_{t+1}})\\ Y_{t}=[\vec{h_{t}},\overset{\leftarrow }{h_{t}}] \end{array}\right.$$

The attention mechanism assigns different weights to parts of the input sequence, helping the model focus on the most important features for the current task. This enhances the model’s ability to understand context and use computational resources more effectively.

First, the similarity between Query and Key is calculated, see Equation 17:

(17) $$e_{ij}=D(q_{i},p_{j})$$

Then, the similarity is normalized with the softmax function, see Equation 18:

(18) $$a_{ij}=\mathrm{softmax}(e_{ij})$$

Finally, the attention weights for each feature sequence at time *t* can be calculated by a weighted sum of its components, as given in Equation 19.

(19) $$c_{i}=\sum_{i=1}^{N}a_{ij}q_{i}$$

In the above equation, the *D* operation represents the dot product function, *q* is the query element, *p* is the element’s address, *e* is the similarity between elements, and *c* is the weight.

In summary, the architecture of the proposed CNN-BiLSTM-Attention hybrid model is illustrated in **Figure 6**. This model sequentially includes an input layer, a convolutional neural network layer, a bidirectional long short-term memory layer, an attention layer, a fully connected layer, and an output layer.

**Figure 6:**
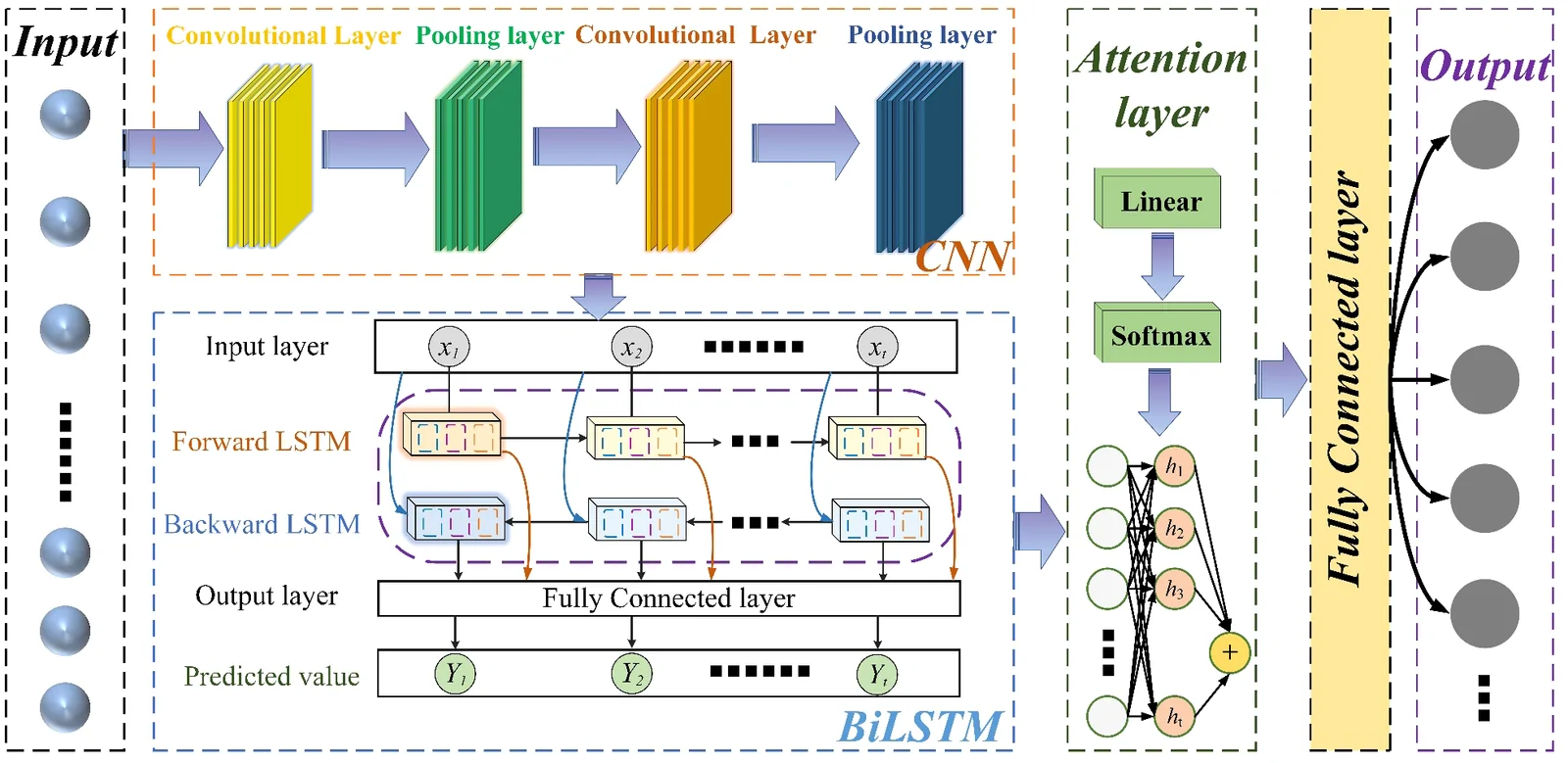
Hybrid network architecture of CNN-BiLSTM incorporating attention mechanism.

### 3.3 IPSO algorithm

Particle Swarm Optimization (PSO) is a swarm intelligence algorithm in evolutionary computation. This algorithm models the solution space as a search space, where particles collaborate to find optimal solutions through iterative updates and information sharing within the population. When better solutions are discovered, the particle swarm moves toward those areas; if improved solutions emerge, the system continues exploring the global optimum through information exchange and state updates. The iterative formulas are shown in Equations 20 and 21.

(20) $$v_{id}^{k+1}=\omega v_{id}^{k}+c_{1}r_{1}(p_{id}^{k}-x_{id}^{k})+c_{2}r_{2}(g_{d}^{k}-x_{id}^{k})$$

(21) $$x_{id}^{k+1}=x_{id}^{k}+v_{id}^{k}$$

In the equations above, *i*=*1*, *2*, *3*, …, *n* and *d*=*1*, *2*, *3*, …, *D*, where denotes the current iteration number of the particle; $v_{id}^{k}$, $p_{id}^{k}$, and $x_{id}^{k}$ represent the *d*-dimensional velocity, individual extremum coordinate, and position component of the *i*-th particle at the *k*-th iteration, respectively; $g_{d}^{k}$ is the *d*-dimensional component in the global optimal solution vector at the *k*-th iteration; *ω* indicates the inertia weight of particle flight velocity, the inertia weight parameters are determined by multiple simulation comparison tests to balance global search ability and convergence speed; *c*_1_ and *c*_2_ denote learning factors; and *r*_1_ and *r*_2_ are random real numbers between (0, 1).

However, this algorithm still has some limitations: particle search mainly relies on its own experience and neighborhood data, which limits its overall perception ability; low population diversity can easily lead to premature convergence, trapping the algorithm in local optima. Although adjusting parameters such as inertia weights and learning rates can help reduce these issues somewhat, it is hard to fundamentally improve the algorithm’s ability to escape local minima.

To address these issues, this study incorporates crossover and mutation operations from genetic algorithms to update the particle swarm archive set, thereby increasing population diversity and improving the algorithm’s ability to avoid local optima. This approach allows for more thorough exploration of particles within the solution space, ultimately enhancing optimization efficiency. The strategy is illustrated in **Figure 7** (Wang et al., 2021).

**Figure 7:**
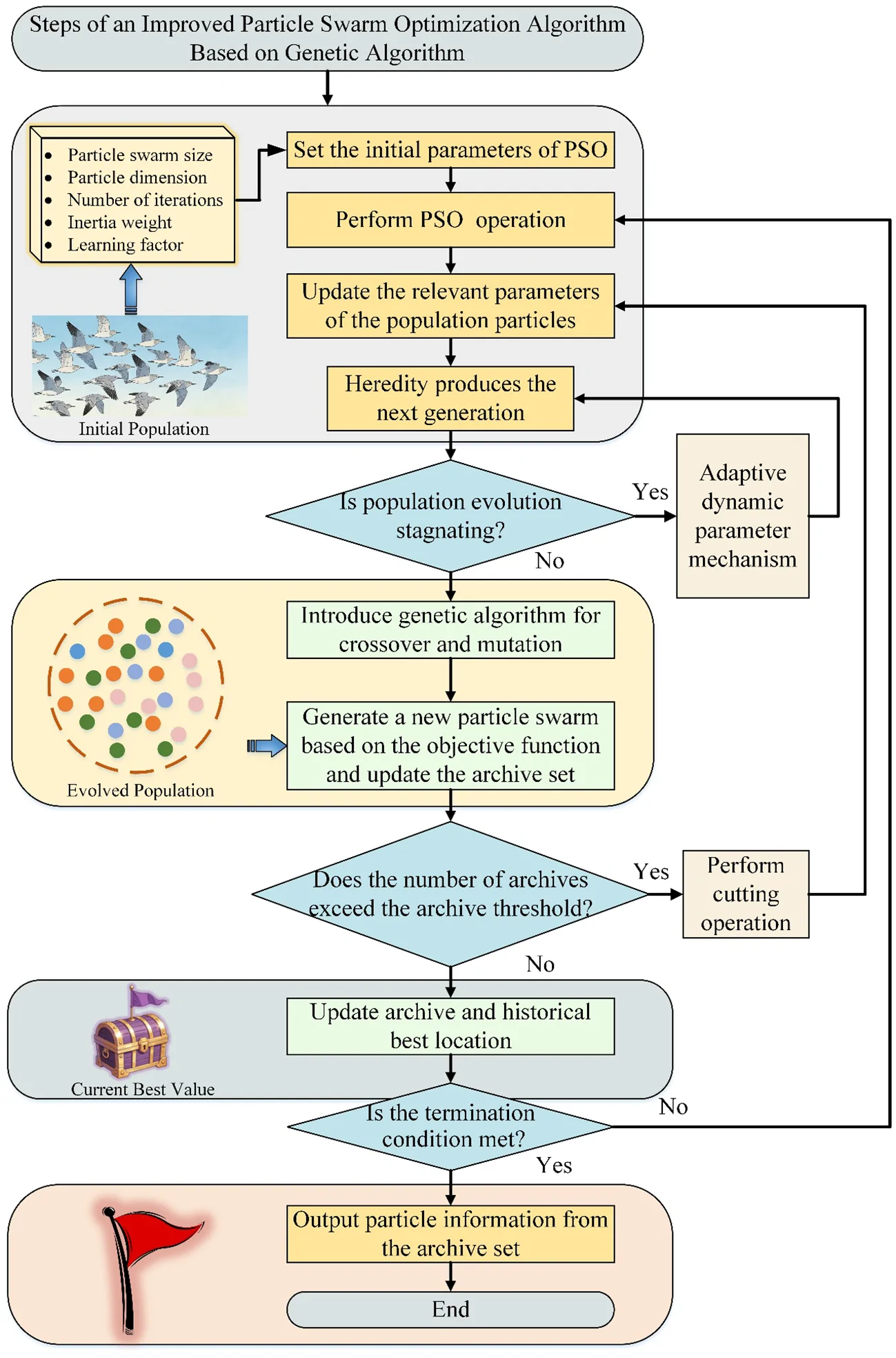
Optimization computation workflow of the improved particle swarm algorithm (IPSO).

### 3.4 Improvement strategies for the UKF algorithm

The principle of improving the UKF algorithm with the CNN-BiLSTM-Attention hybrid model and IPSO algorithm is depicted in **Figure 8**. The main process involves replacing the standard UKF’s state and observation models with those from the CNN-BiLSTM-Attention hybrid model, then performing state prediction, observation prediction, and update steps to progressively refine velocity and attitude estimates.

**Figure 8:**
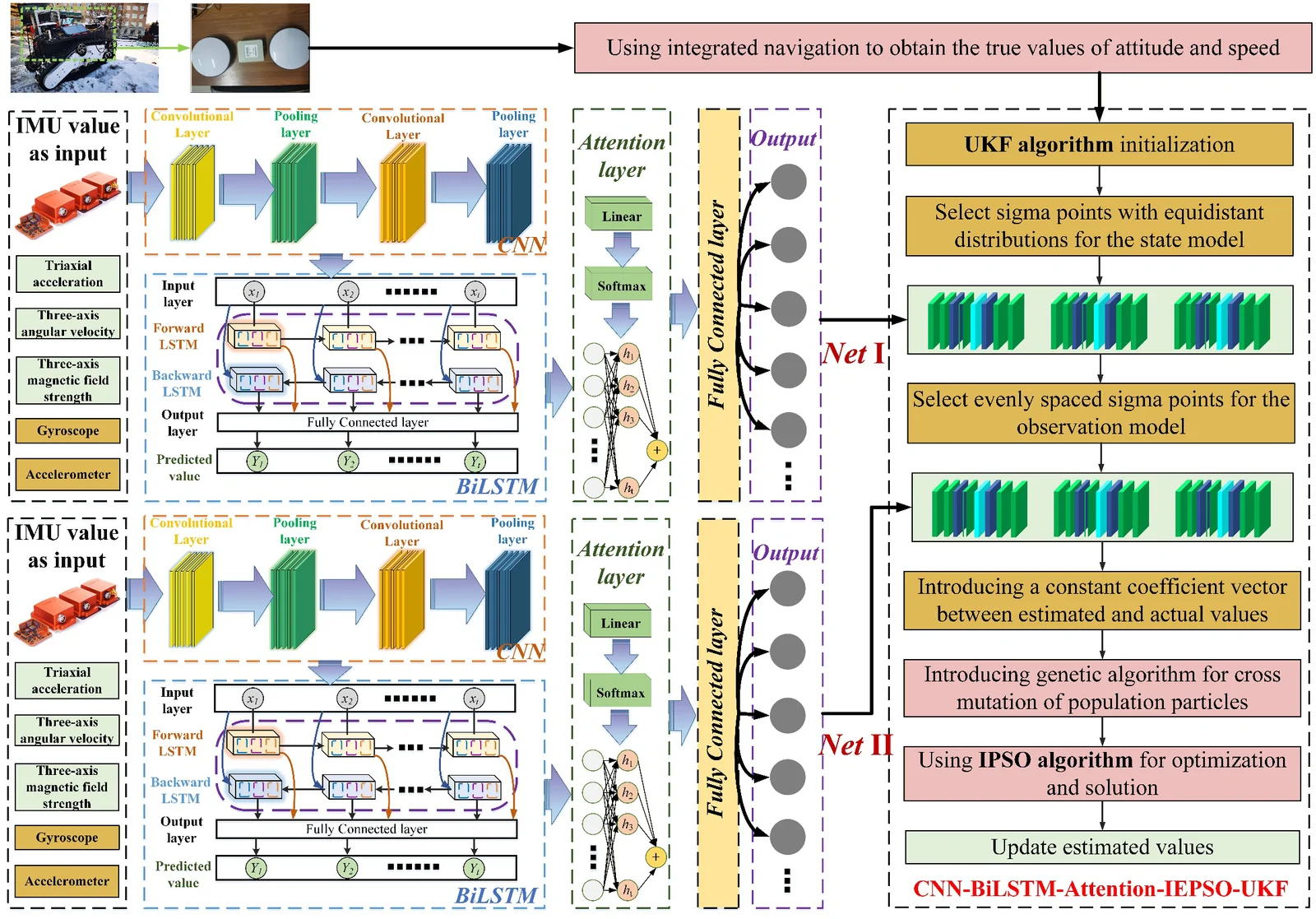
Complete algorithm implementation workflow of the hybrid estimation method.

During the IMU data resolution process, the improved UKF algorithm is employed to resolve attitude and velocity. When establishing the state model using the CNN-BiLSTM-Attention hybrid model, the input vector for the hybrid model is constructed according to the following procedure: The state vector of the carrier platform at time *k* consists of the velocity $t_{k-1}$, acceleration $\left[\begin{array}{ccc} a_{x,k-1} & a_{y,k-1} & a_{z,k-1} \end{array}\right]$, angular velocity $\left[\begin{array}{ccc} \omega_{x,k-1} & \omega_{y,k-1} & \omega_{z,k-1} \end{array}\right]$, magnetic field intensity $\left[\begin{array}{ccc} m_{x,k-1} & m_{y,k-1} & m_{z,k-1} \end{array}\right]$, calculated attitude $\left[\begin{array}{cccc} q_{w,k-1}^{imu} & q_{x,k-1}^{imu} & q_{y,k-1}^{imu} & q_{z,k-1}^{imu} \end{array}\right]$, and velocity $\left[\begin{array}{ccc} v_{x,k-1}^{imu} & v_{y,k-1}^{imu} & v_{z,k-1}^{imu} \end{array}\right]$, totaling 17 dimensions.

The attitude and velocity values obtained from integrated navigation at time *k* are treated as the true values and regarded as the desired output $\left[\begin{array}{ccccccc} q_{w,t}^{k} & q_{x,t}^{k} & q_{y,t}^{k} & q_{z,t}^{k} & v_{x,t}^{k} & v_{y,t}^{k} & v_{z,t}^{k} \end{array}\right]$of the state model. The output vector has 7 dimensions, thus establishing the state model.

Similarly, when using the proposed model to establish the observation model, the input vector for the hybrid model is constructed. The input vector comprises the velocity $t_{k}$, acceleration $\left[\begin{array}{ccc} a_{x,k} & a_{y,k} & a_{z,k} \end{array}\right]$, angular velocity $\left[\begin{array}{ccc} \omega_{x,k} & \omega_{y,k} & \omega_{z,k} \end{array}\right]$, magnetic field intensity $\left[\begin{array}{ccc} m_{x,k} & m_{y,k} & m_{z,k} \end{array}\right]$, calculated attitude $\left[\begin{array}{cccc} q_{w,k}^{imu} & q_{x,k}^{imu} & q_{y,k}^{imu} & q_{z,k}^{imu} \end{array}\right]$, and velocity $\left[\begin{array}{ccc} v_{x,k}^{imu} & v_{y,k}^{imu} & v_{z,k}^{imu} \end{array}\right]$ at time, totaling 17 dimensions.

The attitude and velocity values obtained from integrated navigation at time *k* are treated as the true values and regarded as the desired output $\left[\begin{array}{ccccccc} q_{w,t}^{k} & q_{x,t}^{k} & q_{y,t}^{k} & q_{z,t}^{k} & v_{x,t}^{k} & v_{y,t}^{k} & v_{z,t}^{k} \end{array}\right]$ of the observation model. The output vector has 7 dimensions, thereby establishing the observation model.

The aforementioned state and observation models are denoted *Net*I and *Net*II, respectively. Versus the traditional UKF modeling approach, the improved algorithm replaces the state model *f* (·) with *Net*I, obtaining the state prediction values through the hybrid model, as shown in Equation 22:

(22) $$X_{i,k/k-1}=net\text{I}(\gamma_{i,k-1/k-1})$$

Similarly, when setting up the observation model with the hybrid model, the improved algorithm replaces the observation model *h* (·) with *Net*II, as shown in Equation 23:

(23) $$Y_{i,k/k-1}=net\text{II}(\xi_{i,k/k-1})$$

To reduce modeling errors and estimation biases and to further enhance accuracy, this study introduces a constant-coefficient compensation vector *λ* between the estimated and true values to offset accumulated errors at each step, thereby obtaining corrected attitude and velocity. This compensation vector *λ* must satisfy the condition established in Equation 24.

(24) $$\left[\begin{array}{c} q_{w,t}^{k}\\ q_{x,t}^{k}\\ q_{y,t}^{k}\\ q_{z,t}^{k}\\ v_{x,t}^{k}\\ v_{y,t}^{k}\\ v_{z,t}^{k} \end{array}]=\lambda [\begin{array}{c} {\hat{q}}_{w}^{k}\\ {\hat{q}}_{x}^{k}\\ {\hat{q}}_{y}^{k}\\ {\hat{q}}_{z}^{k}\\ {\hat{v}}_{x}^{k}\\ {\hat{v}}_{y}^{k}\\ {\hat{v}}_{z}^{k} \end{array}]+K_{k}[[\begin{array}{c} q_{w,k-1}^{imu}\\ q_{x,k-1}^{imu}\\ q_{y,k-1}^{imu}\\ q_{z,k-1}^{imu}\\ v_{x,k}^{imu}\\ v_{y,k}^{imu}\\ v_{z,k}^{imu} \end{array}]-[\begin{array}{c} {\hat{q}}_{w,k-1}^{imu}\\ {\hat{q}}_{x,k-1}^{imu}\\ {\hat{q}}_{y,k-1}^{imu}\\ {\hat{q}}_{z,k-1}^{imu}\\ {\hat{v}}_{x,k}^{imu}\\ {\hat{v}}_{y,k}^{imu}\\ {\hat{v}}_{z,k}^{imu} \end{array}]\right]$$

In Equation 24, only λ remains unknown, which allows the problem of solving the equation to be transformed into an optimization problem that can be addressed using the IPSO algorithm. The parameters for IMU state estimation include seven dimensions: three-axis attitude and velocity (before quaternion conversion). Therefore, *λ* takes the form of a vector $\tilde{\lambda }=\{\lambda_{1},\lambda_{2},\lambda_{3},\lambda_{4},\lambda_{5},\lambda_{6},\lambda_{7}\}$, with positive and negative values indicating the directions of attitude and velocity, respectively, within the following range of values, as expressed in Equation 25:

(25) $$-0.8\leq \lambda_{1},\lambda_{2},\lambda_{3},\lambda_{4},\lambda_{5},\lambda_{6},\lambda_{7}\leq 1.2\cup 0.8\leq \lambda_{1},\lambda_{2},\lambda_{3},\lambda_{4},\lambda_{5},\lambda_{6},\lambda_{7}\leq 1$$

To enable the introduced constant coefficient compensation to effectively minimize the deviation between the estimated values and the true values, this paper defines the following objective function, as given in Equation 26:

(26) $$\min F_{obj}=[\begin{array}{c} q_{w,t}^{k}\\ q_{x,t}^{k}\\ q_{y,t}^{k}\\ q_{z,t}^{k}\\ v_{x,t}^{k}\\ v_{y,t}^{k}\\ v_{z,t}^{k} \end{array}]-[\begin{array}{c} {\hat{q}}_{w}^{k}\\ {\hat{q}}_{x}^{k}\\ {\hat{q}}_{y}^{k}\\ {\hat{q}}_{z}^{k}\\ {\hat{v}}_{x}^{k}\\ {\hat{v}}_{y}^{k}\\ {\hat{v}}_{z}^{k} \end{array}]-K_{k}[[\begin{array}{c} q_{w,k-1}^{imu}\\ q_{x,k-1}^{imu}\\ q_{y,k-1}^{imu}\\ q_{z,k-1}^{imu}\\ v_{x,k}^{imu}\\ v_{y,k}^{imu}\\ v_{z,k}^{imu} \end{array}]-[\begin{array}{c} {\hat{q}}_{w,k-1}^{imu}\\ {\hat{q}}_{x,k-1}^{imu}\\ {\hat{q}}_{y,k-1}^{imu}\\ {\hat{q}}_{z,k-1}^{imu}\\ {\hat{v}}_{x,k}^{imu}\\ {\hat{v}}_{y,k}^{imu}\\ {\hat{v}}_{z,k}^{imu} \end{array}]]$$

After finding the optimal value of the constant coefficient vector $\tilde{\lambda }=\{\lambda_{1},\lambda_{2},\lambda_{3},\lambda_{4},\lambda_{5},\lambda_{6},\lambda_{7}\}$, the overall estimated value is given in Equation 27:

(27) $$\left[\begin{array}{c} q_{w,\lambda }^{k}\\ q_{x,\lambda }^{k}\\ q_{y,\lambda }^{k}\\ q_{z,\lambda }^{k}\\ v_{x,\lambda }^{k}\\ v_{y,\lambda }^{k}\\ v_{z,\lambda }^{k} \end{array}]=\tilde{\lambda }[\begin{array}{c} {\hat{q}}_{w}^{k}\\ {\hat{q}}_{x}^{k}\\ {\hat{q}}_{y}^{k}\\ {\hat{q}}_{z}^{k}\\ {\hat{v}}_{x}^{k}\\ {\hat{v}}_{y}^{k}\\ {\hat{v}}_{z}^{k} \end{array}]+K_{k}[[\begin{array}{c} q_{w,k-1}^{imu}\\ q_{x,k-1}^{imu}\\ q_{y,k-1}^{imu}\\ q_{z,k-1}^{imu}\\ v_{x,k}^{imu}\\ v_{y,k}^{imu}\\ v_{z,k}^{imu} \end{array}]-[\begin{array}{c} {\hat{q}}_{w,k-1}^{imu}\\ {\hat{q}}_{x,k-1}^{imu}\\ {\hat{q}}_{y,k-1}^{imu}\\ {\hat{q}}_{z,k-1}^{imu}\\ {\hat{v}}_{x,k}^{imu}\\ {\hat{v}}_{y,k}^{imu}\\ {\hat{v}}_{z,k}^{imu} \end{array}]\right]$$

In summary, the improved dynamic parameter estimation algorithm framework can be divided into three main parts: first, using the CNN-BiLSTM-Attention hybrid model to replace the state and observation models within the UKF algorithm; second, measuring the residuals between the improved UKF estimates and true values, then applying the enhanced PSO algorithm to perform worldwide optimization of the constant-coefficient compensation vector for the correction model; finally, reinitializing the UKF algorithm, and within this reinitialized architecture, combining deep learning prediction outputs with optimized constant-coefficient compensation terms to ultimately produce updated attitude and velocity estimates.

## 4 Simulation experiments

### 4.1 Experimental configuration

This section confirms the effectiveness of the proposed algorithm and compares it with popular deep learning networks. The experimental setup uses a custom-designed tracked agricultural transport platform, with key technical parameters listed in **Table 1** (Wang et al., 2024).

**Table 1:** Key technical parameters of the experimental platform.

Parameter	Symbol	Unit	Numerical value
Total mass of the platform	*M*	Kg	200
Track shoe width	*b*	m	0.18
Width of the platform	*D*	m	0.38
Total length of the track chassis	*L_1_*	m	1.2
Track grounding length	*l*	m	0.85
Track Height	*H_1_*	m	0.3
Track tension coefficient	*n_k_*	–	0.35
Reduction ratio	*i*	–	100
Ground pressure ratio	*p*	Mpa	0.045

To develop a high-quality, data-driven model, this study collected 20, 000 raw datasets from real-world environments and selected 18, 000 valid samples after preprocessing for training and testing. The samples include center-of-mass motion state parameters of the transport platform and synchronized sensor observation data. Among them, 18000 samples were randomly divided into a training set (70%), a validation set (15%), and a testing set (15%), and we used a waiting validation method for model evaluation.

The experimental computing platform is set up as follows: the hardware includes an Intel Core i7-13700H processor, an NVIDIA RTX A500 graphics card, and 48 GB of memory. The software architecture relies on a Python 3.6 environment, integrated with deep learning frameworks such as PyTorch, Keras 2.3.1, and TensorFlow 1.15.0. The experimental workflow is shown in **Figure 9**.

**Figure 9:**
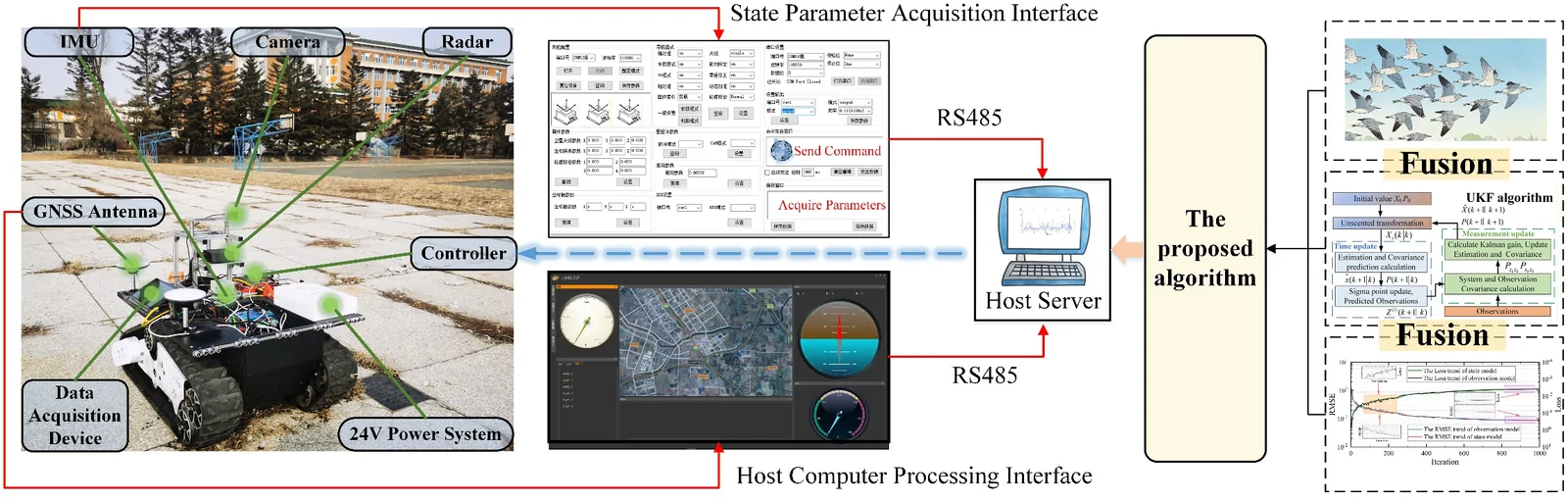
System deployment and data acquisition workflow in field experimental environments.

### 4.2 Optimization solution for constant-coefficient compensation vector

To find the global optimal solution for the compensation vector, this study employs an improved particle swarm optimization algorithm for parameter tuning. To improve convergence and efficiency, key hyperparameters of the IPSO are carefully set, with maximum search velocity limits added to prevent blind searches. Initial parameters are randomly generated within the predefined constrained space to ensure population diversity. The algorithm’s parameter settings are detailed in **Table 2**.

**Table 2:** Parameter configurations of the IPSO algorithm.

Parameter	Parameter value	Parameter	Parameter value
Population size *n_pop_*	100	Crossover probability *Pc*	0.85
Learning factor *c*_1_, *c*_2_	1.5	Number of iterations *n_iter_*	300
Maximum search velocity *V*_max_	1	Minimum search velocity *V*_min_	-1
Inertia weight	0.4~0.8	Mutation rate *P_m_*	0.01

To ensure optimization accuracy, the IPSO algorithm is considered to have converged when the iterative deviation of the design variables or the objective function value falls below 10^−4^. **Figures 10**, **11** show the dynamic evolution of each constant-coefficient variable and the objective function during the optimization process, respectively. The results indicate that all parameters converge steadily toward optimal solutions as the number of iterations increases, confirming the algorithm’s effectiveness.

**Figure 10:**
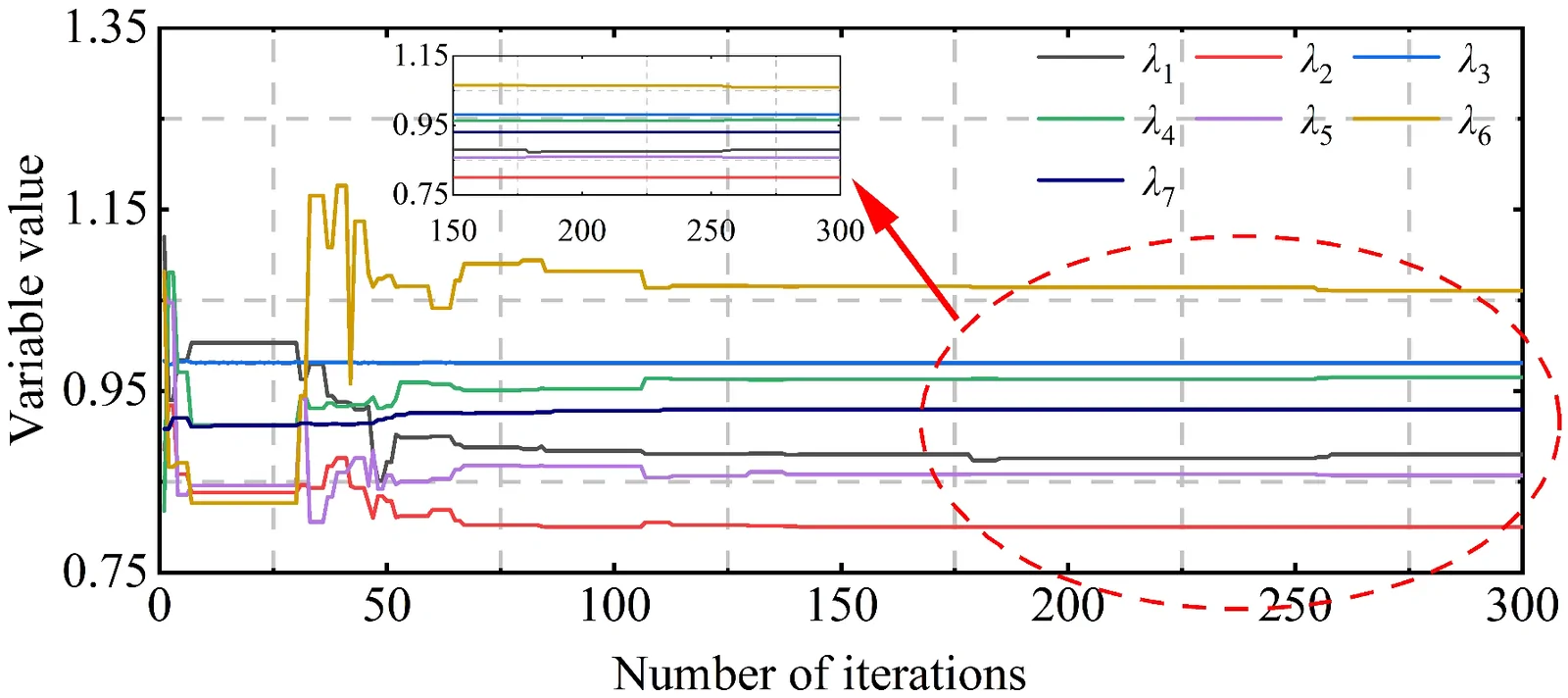
IPSO optimization process for key design variables.

**Figure 11:**
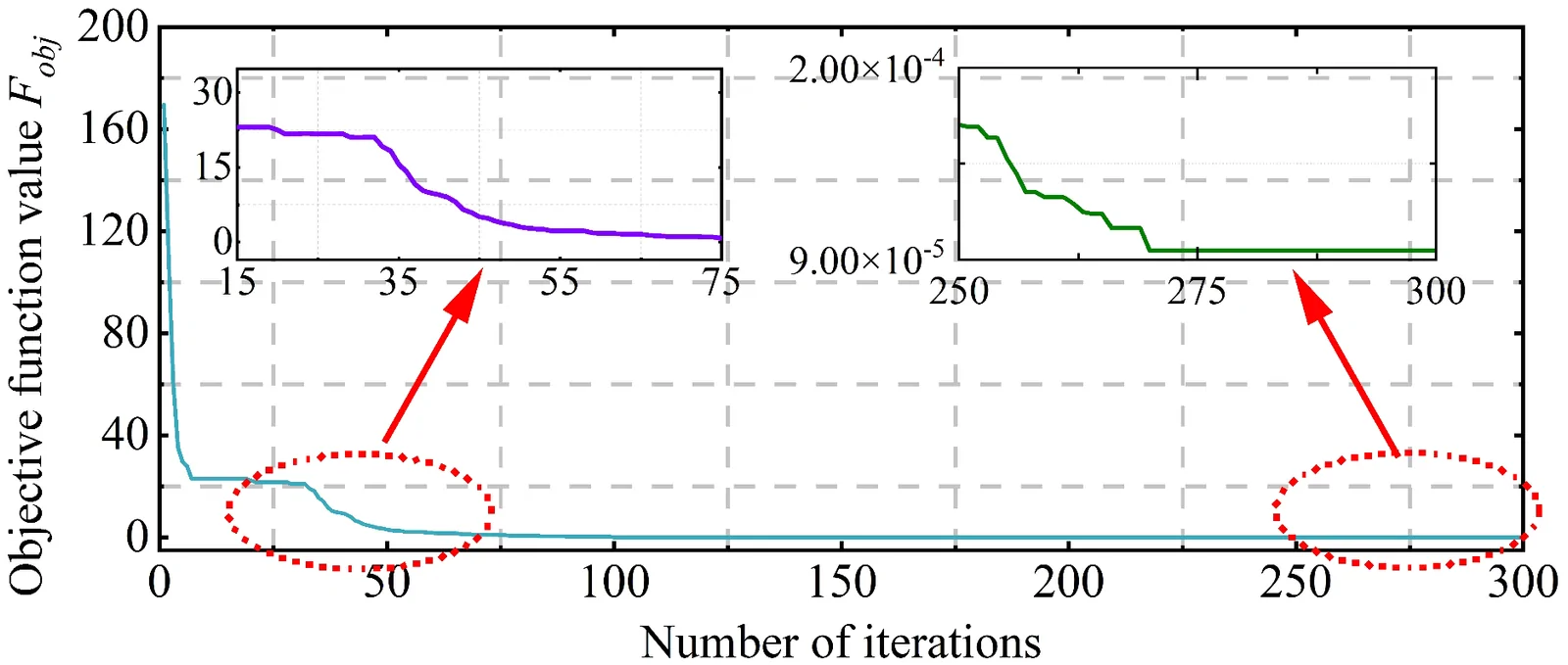
Convergence curve of objective function values versus iteration numbers.

As shown in **Figure 11**, the objective function value *Fobj* of the IPSO algorithm stabilizes after 300 iterations, satisfying the preset convergence condition. **Table 3** presents the optimal estimated values of the constant-coefficient compensation vector obtained at this point. Therefore, this study defines the compensation vector as to address dynamic parameter estimation errors.

**Table 3:** Optimal parameter configuration based on the IPSO algorithm.

Parameters	*λ*_1_	*λ*_2_	*λ*_3_	*λ*_4_	*λ*_5_	*λ*_6_	*λ*_7_
Optimized Value	0.88	0.85	0.99	0.97	0.86	1	0.93

### 4.3 Results of CNN-BiLSTM-attention

In the CNN-BiLSTM-Attention-IPSO-UKF algorithm framework, the CNN-BiLSTM-Attention hybrid model is used to build the state and observation models, respectively. The training involves 1000 iterations with an initial learning rate of 0.01, using the Adam optimizer, and the Root Mean Square Error (RMSE) serving as the regression objective function. To reduce overfitting, a Dropout layer with a rate of 0.25 is added after the BiLSTM layer, followed by a fully connected layer to produce the output.

We input the acceleration, angular velocity, magnetic field strength, and computed attitude and velocity data collected at time step *k-1* into the hybrid model. The expected output of the state model is labeled with the actual attitude and velocity values collected by the integrated navigation system at time step *k*, thereby establishing a mapping relationship from input to output. The trends in the root mean square error (RMSE) and loss function (Loss) during training are shown in **Figure 12**.

**Figure 12:**
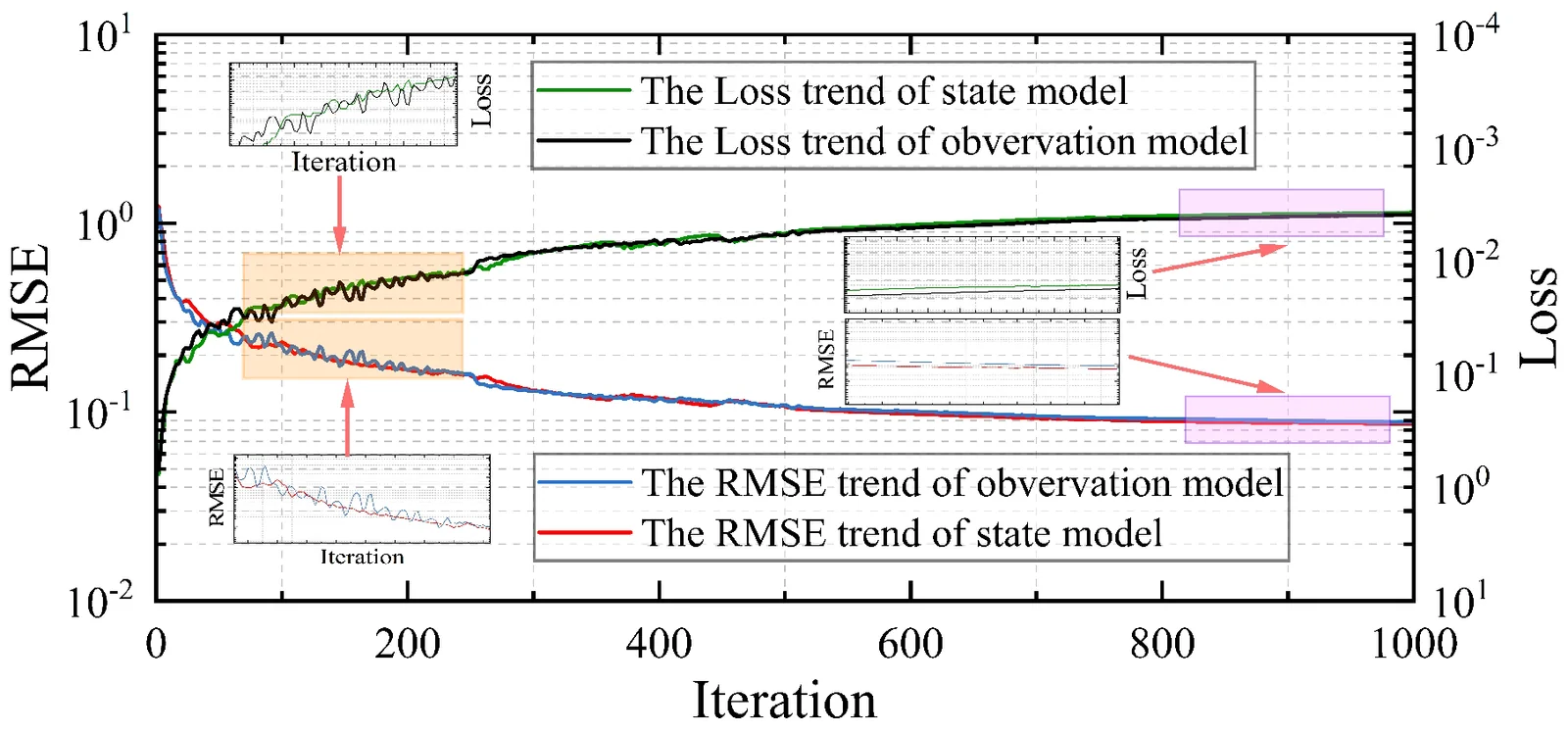
Joint training process of state estimation network and observation correction network.

Both metrics decrease gradually with increasing iterations, indicating stable model training. Since both RMSE and Loss decrease as iterations progress, the RMSE vertical axis (left side) in the figure is scaled from bottom to top to facilitate intuitive visualization. RMSE converges to a low level quickly, demonstrating the model’s high learning efficiency.

**Figure 12** illustrates the model training process. The Loss and RMSE curves level off after approximately 350 iterations, indicating model convergence. Quantitative evaluation results show that the state and observation models, based on the CNN-BiLSTM-Attention hybrid model, perform well, with *R*² coefficients of 0.998 and 0.997, respectively, and RMSE values of 0.021. These findings demonstrate that the model has very high fitting accuracy for the training data.

An analysis of declining loss functions and RMSE during training, along with high *R*² and low RMSE scores, and the progression of the convergence curve shows that the model remained consistent during both training and validation. This effectively minimized the risks of overfitting and underfitting. These findings confirm that the hybrid model achieves high accuracy and strong generalization in dynamic parameter estimation for tracked agricultural transport platforms.

### 4.4 Comparison with other models

To validate the proposed model, CNN, LSTM, and BiLSTM were adopted as baselines. A comparative analysis was performed across three measures: relative error, mean relative error, and coefficient of determination *R*². The results are shown in **Figure 13**; **Table 4**. (All compared baseline models adopt identical hyperparameters such as learning rate, batch size and training epochs to guarantee fair comparison).

**Figure 13:**
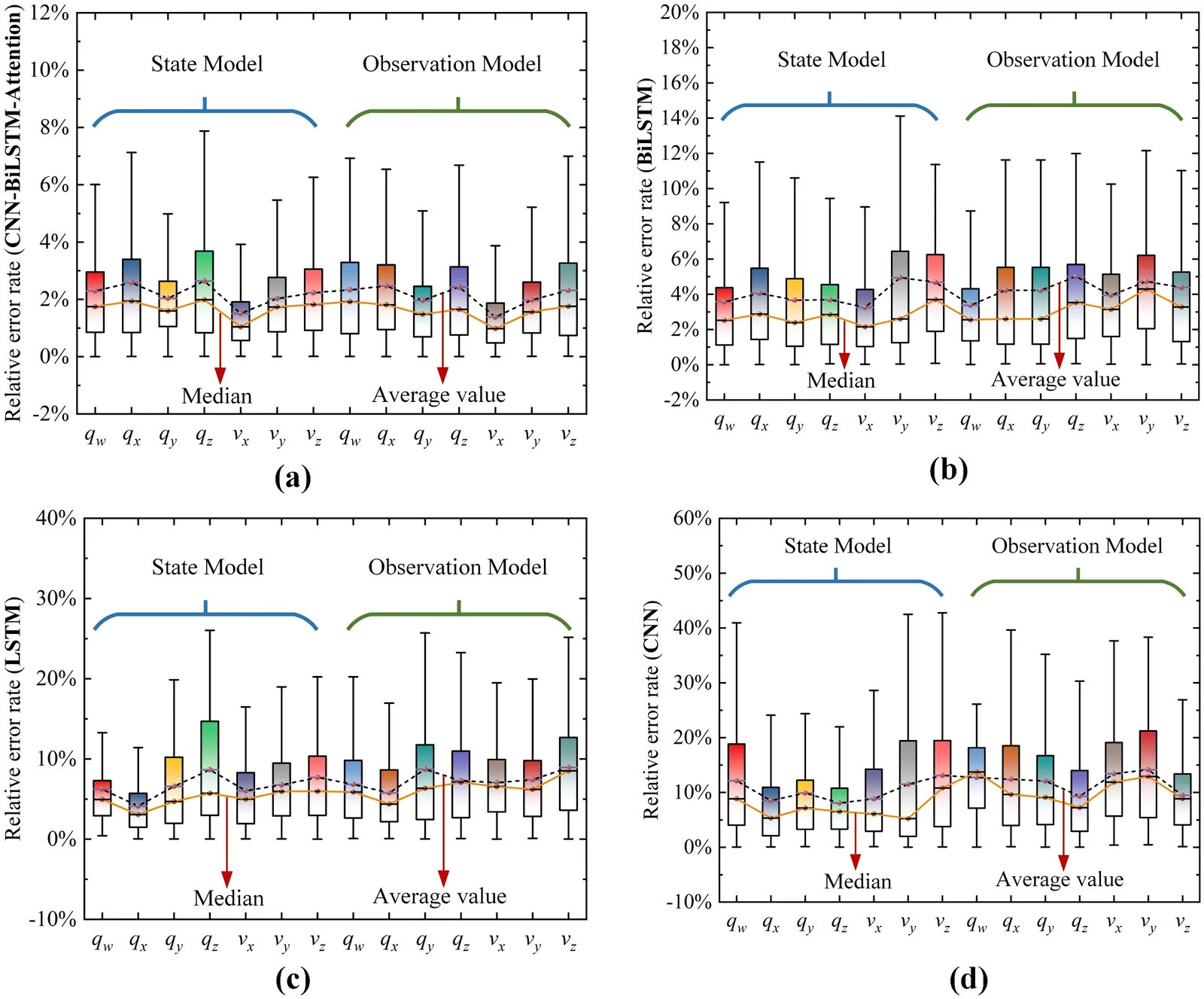
Comparison of relative errors among different network architectures. **(a)** Relative error rate (CNN-BiLSTM-Attention). **(b)** Relative error rate (BiLSTM). **(c)** Relative error rate (LSTM). **(d)** Relative error rate (CNN).

**Table 4:** Comparison of mean relative errors among different network architectures.

Mean relative error (%)									Mean (%)
State model	Base Model	*q_w_*	*q_x_*	*q_y_*	*q_z_*	*v_x_*	*v_y_*	*v_z_*	
	Proposed Model	2.33	2.47	1.96	2.43	1.50	1.98	2.31	2.14
	BiLSTM	3.57	4.06	3.67	3.69	3.25	4.95	4.68	3.98
	LSTM	6.23	4.13	6.58	8.65	6.04	6.76	7.70	6.58
	CNN	12.13	8.54	9.89	8.09	9.03	11.49	13.10	10.32
Observation model	Base Model	*q_w_*	*q_x_*	*q_y_*	*q_z_*	*v_x_*	*v_y_*	*v_z_*	
	Proposed Model	2.30	2.58	2.03	2.64	1.38	2.03	2.23	2.17
	BiLSTM	3.37	4.22	3.71	5.01	3.94	4.73	4.39	4.20
	LSTM	6.83	5.76	8.71	7.30	7.05	7.41	8.94	7.43
	CNN	12.73	12.40	12.10	9.32	13.54	14.14	9.48	11.96

The proposed model significantly outperforms the baseline in both state and observation model fitting. For the state model, the CNN achieved a mean relative error of 10.32%, suggesting limited feature extraction. BiLSTM and LSTM networks showed errors above 3% and 6%, both higher than the hybrid model. The proposed model reduced the mean relative error in state model fitting by 8.18% compared to CNN, by 1.84% compared to BiLSTM, and by 4.44% compared to LSTM. In observation model fitting, it achieved a mean relative error of 2.17%, showing an improvement of 9.79% over CNN, 2.03% over BiLSTM, and 5.26% over LSTM. These results confirm its accuracy and stability in parameter fitting.

As shown in **Figure 14**, the proposed hybrid model attains significantly higher determination coefficients *R*² than the baseline network, with improvements of 1.13%, 4.82%, and 10.21% over the BiLSTM, LSTM, and CNN networks, respectively. This indicates greater explanatory power for the target variable’s variance and a better goodness-of-fit, confirming the model’s advantage in capturing the input-output mapping relationship.

**Figure 14:**
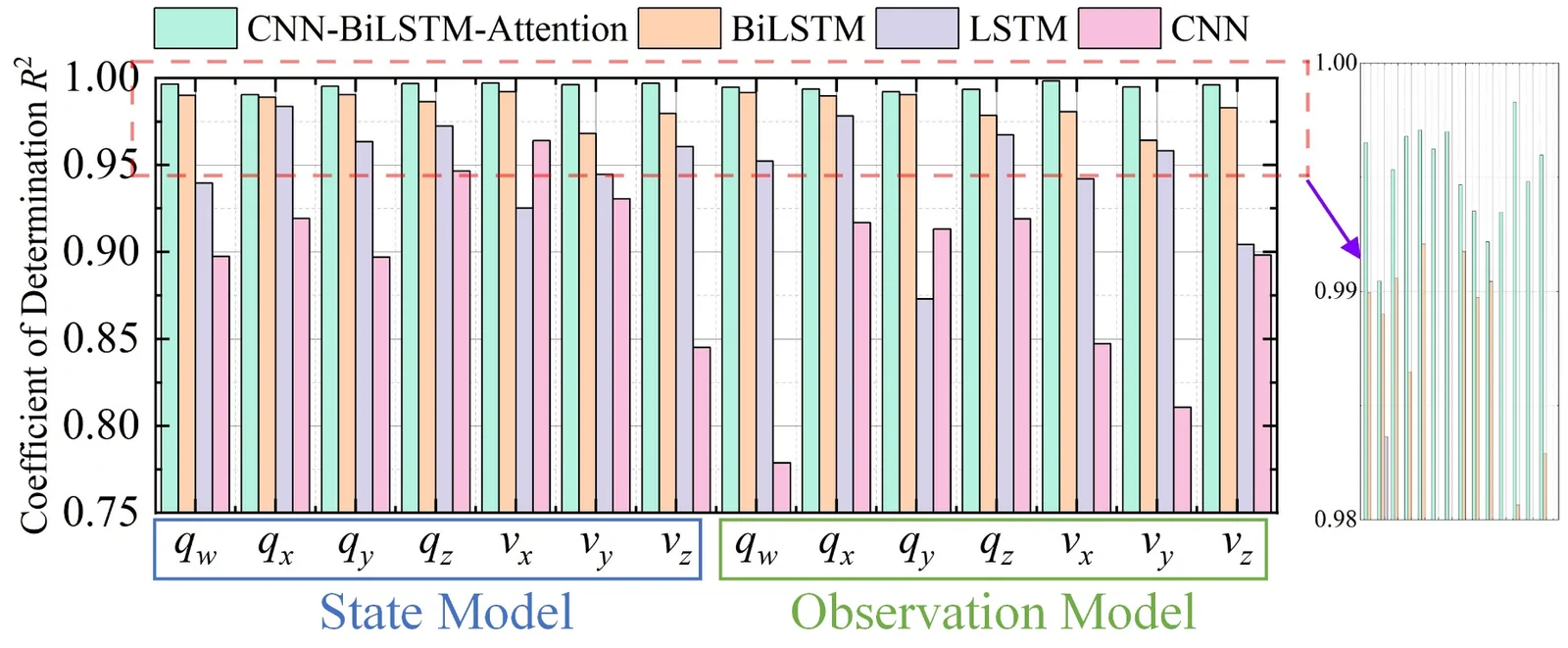
Comparison of R2 between the hybrid model and baseline networks.

## 5 Ground experimental results and discussion

### 5.1 Experimental preparation

To further assess the algorithm’s estimation performance under real-world conditions, this study performed circular driving tests using a physical prototype. Circular driving is a common test for evaluating handling stability in agricultural machinery, as moderate speeds usually induce body roll; it is extensively used for developing and validating stability control algorithms. During the tests, platform attitude and velocity were monitored and recorded in real time, with the planned trajectory shown in **Figure 15**.

**Figure 15:**
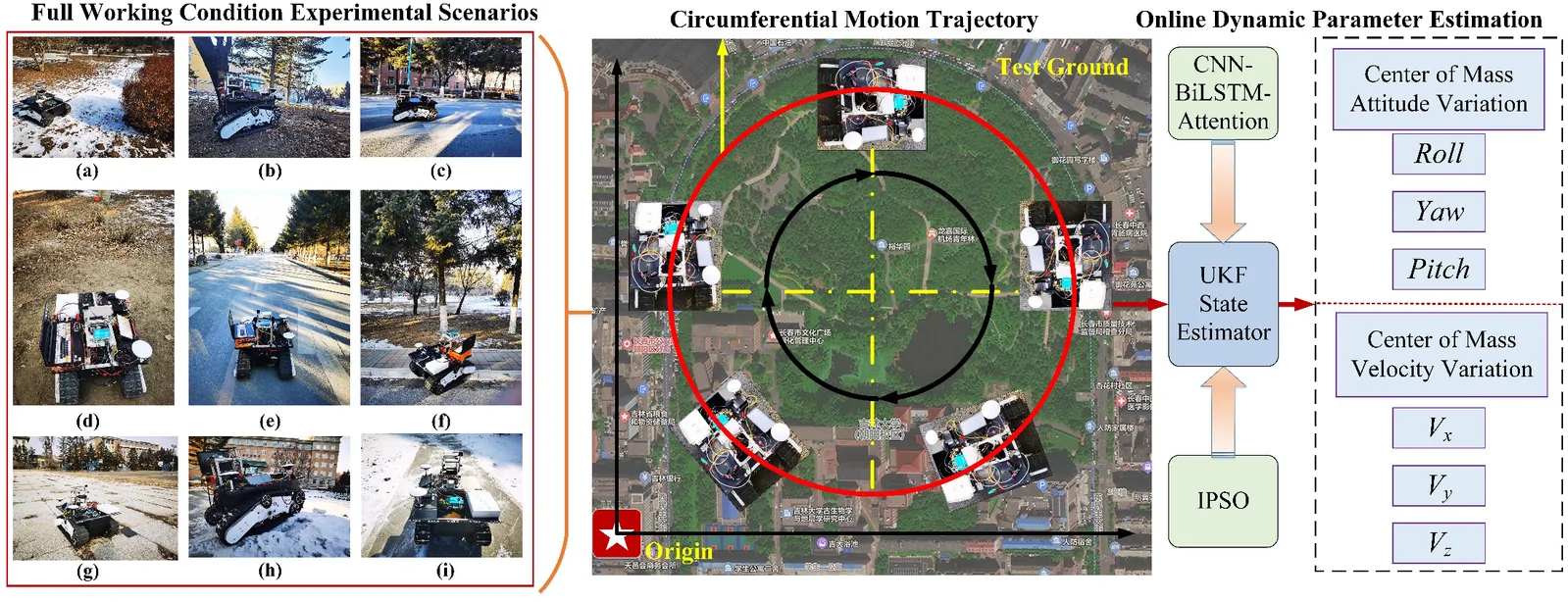
Real-vehicle circular trajectory experimental workflow, **(a–i)** Ground experimental scenarios.

### 5.2 Experimental error analysis

To validate the accuracy and reliability of the proposed hybrid algorithm, an inertial measurement unit (IMU) was installed at the platform’s center of mass. The dynamic information of the platform’s center of mass (including attitude and velocity) output by the IMU served as the reference ground truth. The performance of the proposed algorithm was evaluated through comparative experiments against the Unscented Kalman Filter (UKF), Adaptive Volume Kalman Filter (ACKF, which is an improved volumetric Kalman algorithm that can adaptively adjust noise covariance online. Quan et al., 2026), and Long Short-Term Memory-based Improved Unscented Kalman Filter (LSTM-IPSO-UKF). Additionally, Equations 28, 29 provide the parameters for the system process noise covariance matrix *Q* and the measurement noise covariance matrix *R*:

(28) $$Q=\text{diag}[1\times 10^{4};1\times 10^{-4};1\times 10^{-5};1\times 10^{4};1\times 10^{-6};1\times 10^{-6};1\times 10^{-4}]$$

(29) $$R=\text{diag}[1\times 10^{4};1\times 10^{-4};1\times 10^{4};1\times 10^{-4};1\times 10^{-4};1\times 10^{-4};1\times 10^{4}]$$

To quantitatively assess the experimental results, this study uses Mean Absolute Error (MAE) and Root Mean Square Error (RMSE) as evaluation metrics, with their calculation formulas given in Equations 30 and 31.

(30) $$\text{MAE}=\frac{1}{N}\sum_{i=1}^{N}\left|{\hat{x}}_{i,k}-x_{i,k}\right|$$

(31) $$\text{RMSE}=\sqrt{\frac{1}{N}\sum_{i=1}^{N}{\left({\hat{x}}_{i,\text{k}}-x_{i,k}\right)}^{2}}$$

In the equations above, $x_{i,k}$ and ${\hat{x}}_{i,k}$ represent the true value obtained from ground experiments and the estimated value computed by the algorithm for the *i*-th dynamic parameter at time *k*, respectively; *N* indicates the number of sampling points.

### 5.3 Ground experimental results

**Figures 16**–**21** present the estimation results and error comparisons for four algorithms of the transport platform’s attitude and velocity under circular driving conditions. The black curves indicate the ground-truth experimental values. In contrast, the red, blue, green, and yellow curves represent the estimation results of LSTM-IPSO-UKF, ACKF, UKF, and the proposed algorithm, respectively.

**Figure 16:**
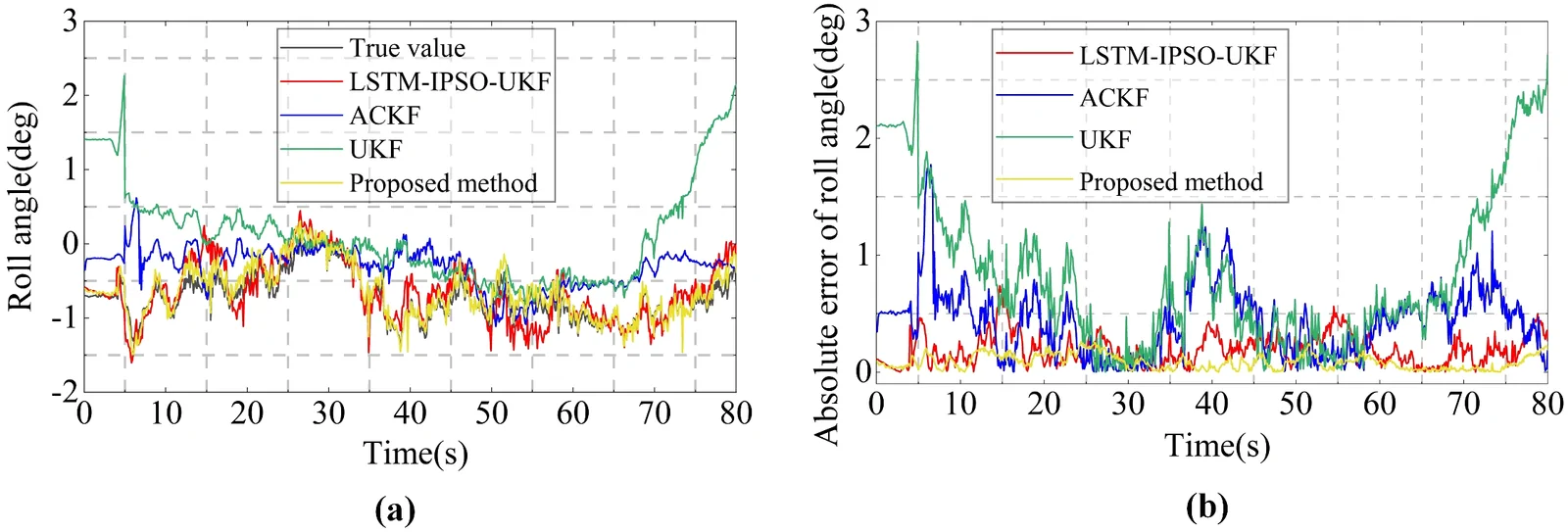
Roll angle estimation results and error analysis. **(a)** Roll angle estimated values. **(b)** Ab-solute error comparison among different algorithms.

**Figure 17:**
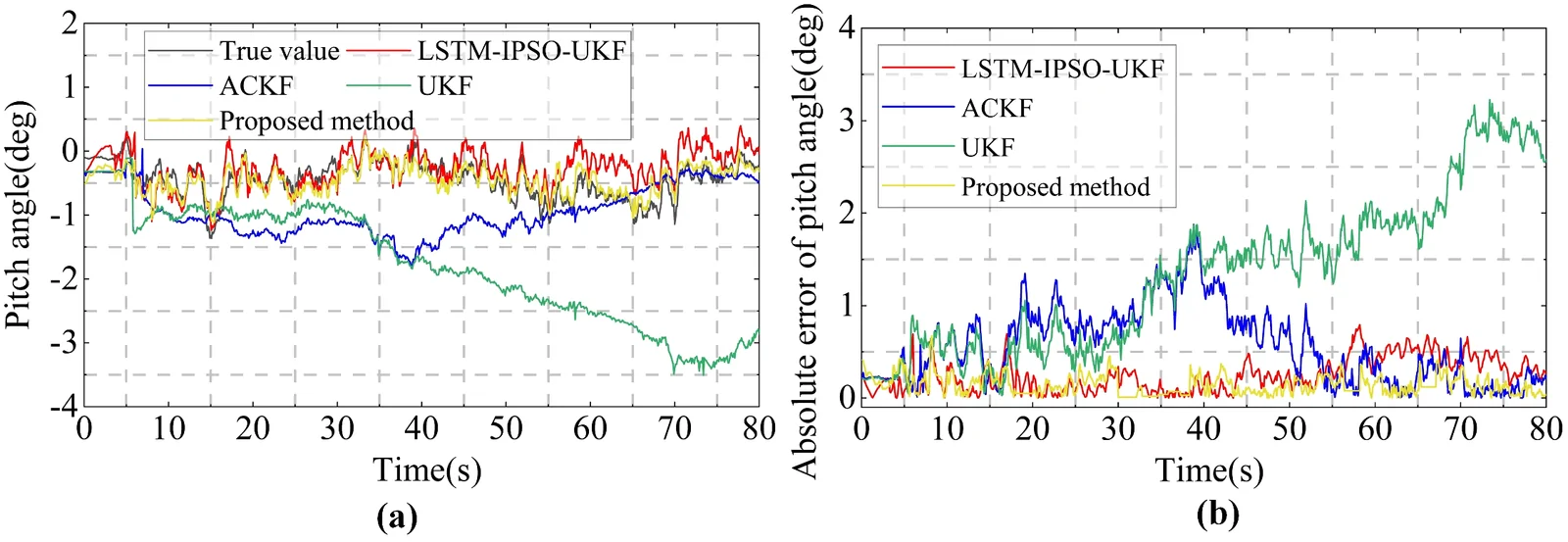
Pitch angle estimation results and error analysis. **(a)** Pitch angle estimated values. **(b)** Absolute error comparison among different algorithms.

**Figure 18:**
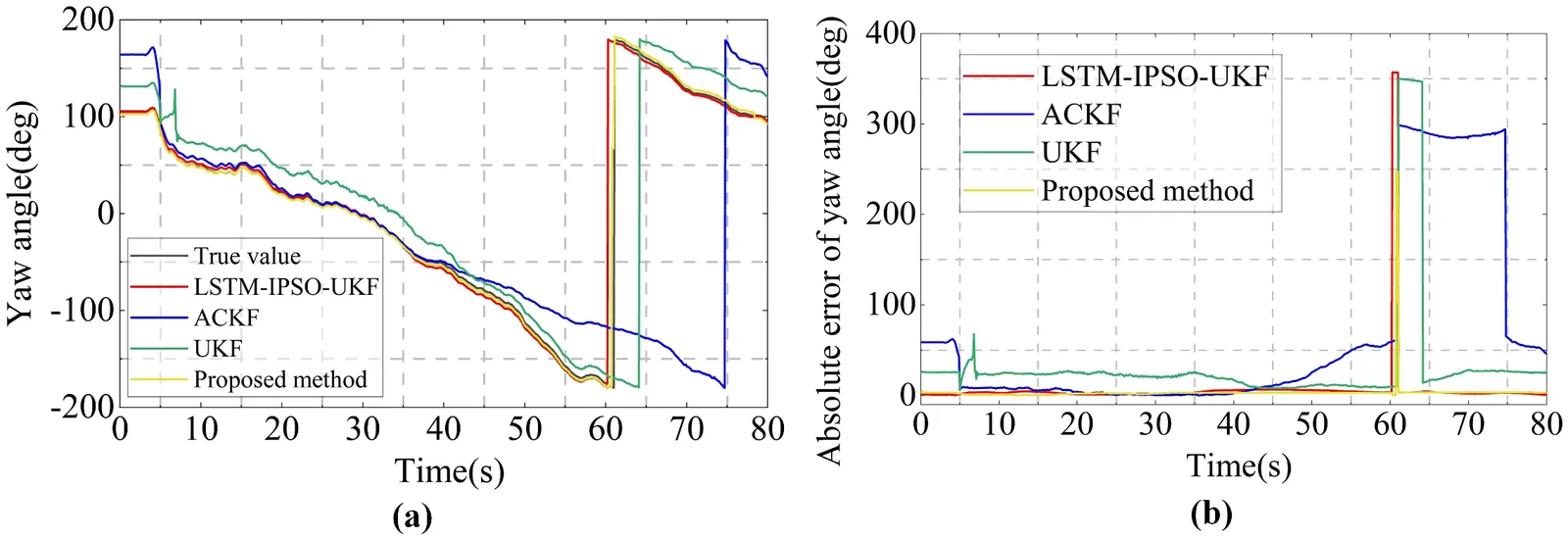
Heading angle estimation results and error analysis. **(a)** Heading angle estimated values. **(b)** Absolute error comparison among different algorithms.

**Figure 19:**
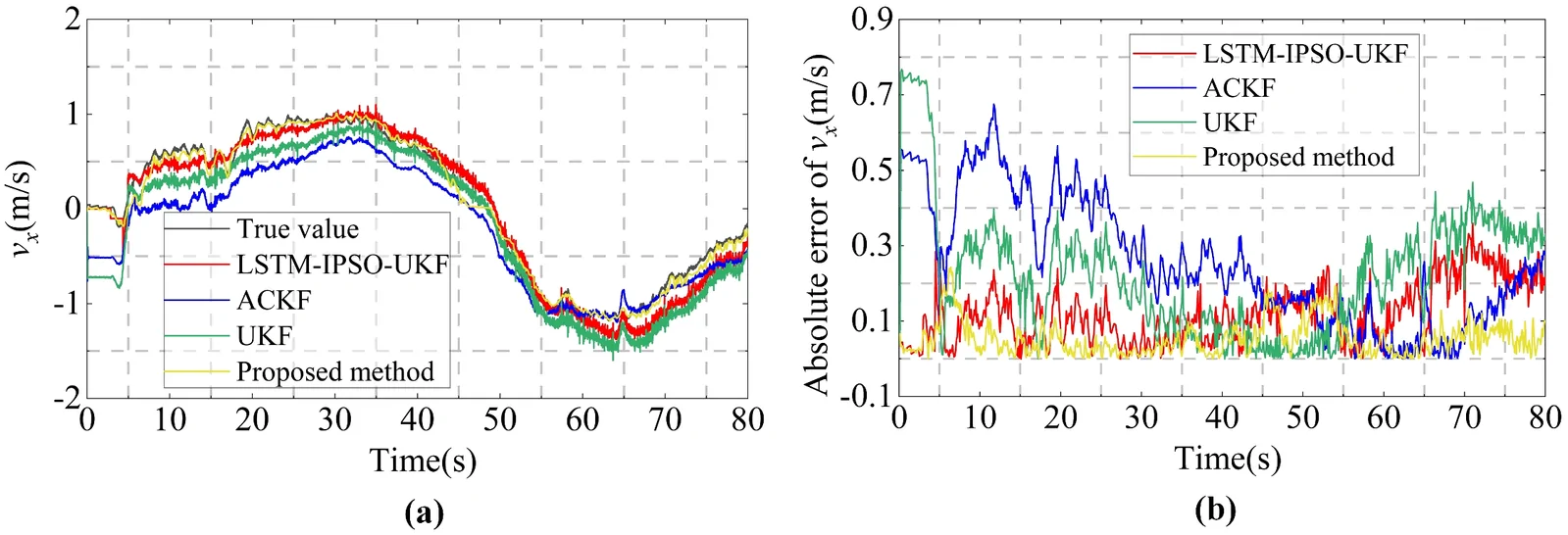
*v_x_* estimation results and error analysis. **(a)** *v_x_* estimated values. **(b)** Absolute error comparison among different algorithms.

**Figure 20:**
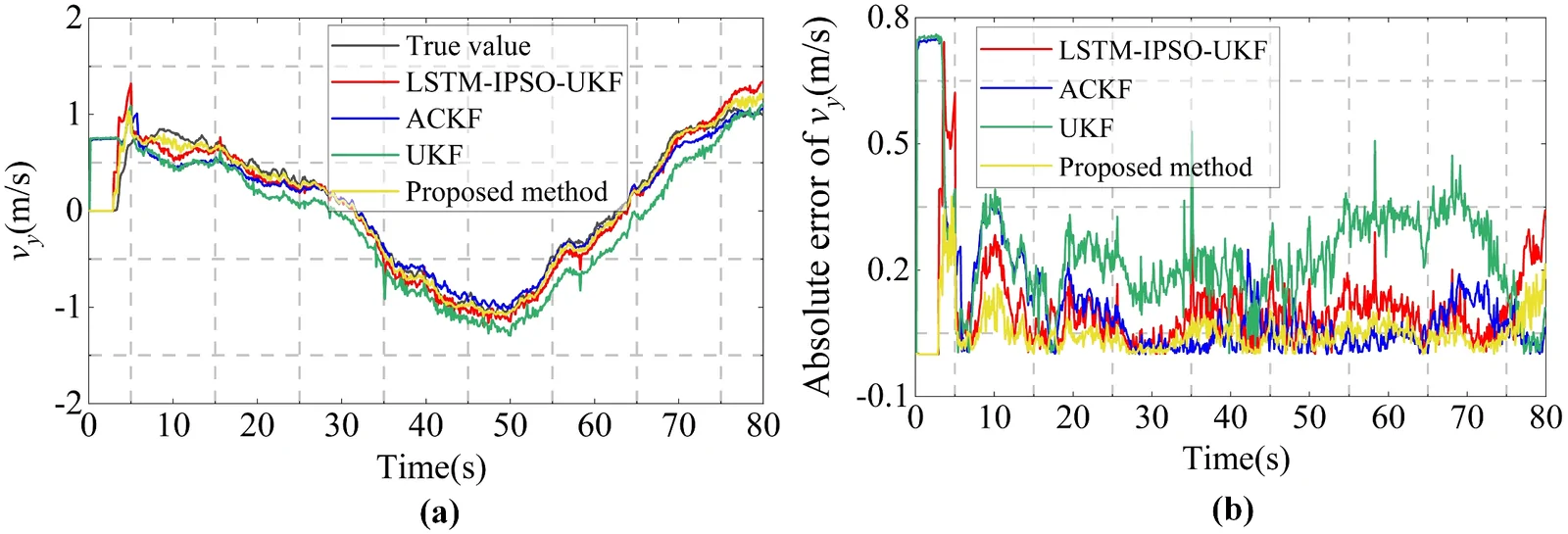
*v_y_* estimation results and error analysis. **(a)** *v_y_* estimated values. **(b)** Absolute error comparison among different algorithms.

**Figure 21:**
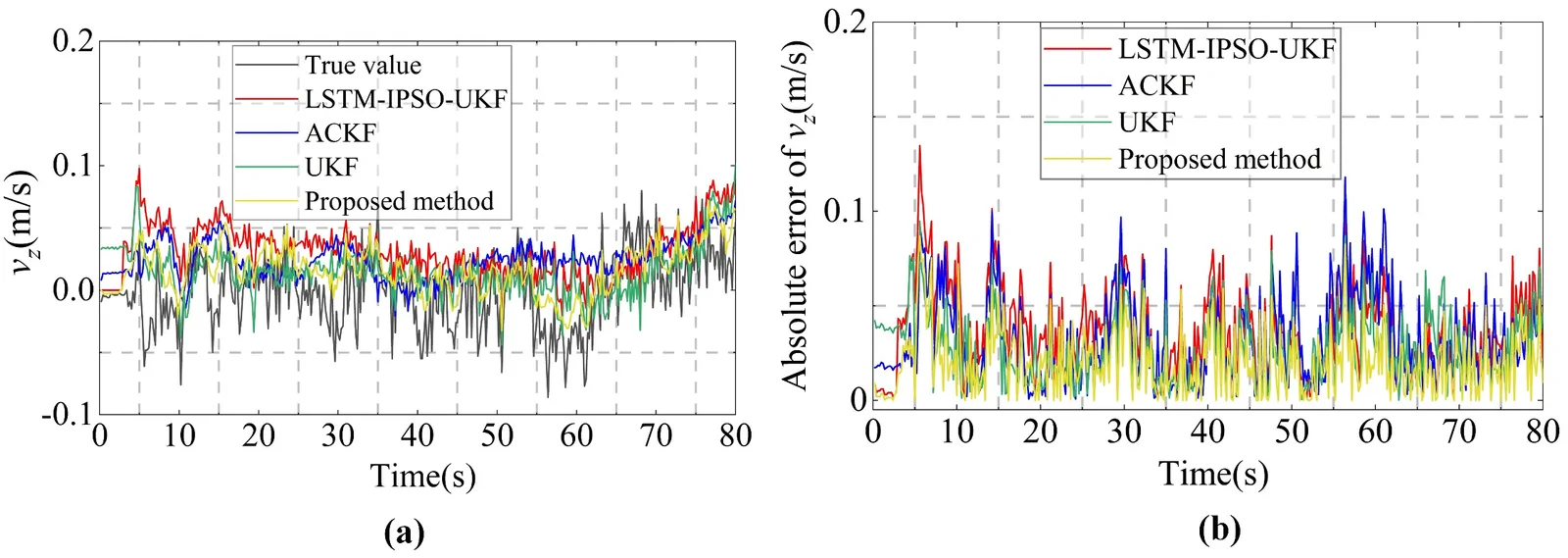
*v_z_* estimation results and error analysis. **(a)** *v_z_* estimated values. **(b)** Absolute error comparison among different algorithms.

#### 5.3.1 Attitude parameter estimation results

As shown in **Figure 16**, compared to the LSTM-IPSO-UKF, ACKF, and UKF algorithms, the proposed algorithm maintains a consistently small roll angle estimation error, demonstrating better accuracy. Specifically, the UKF and ACKF algorithms exhibit large initial errors and tend to diverge over time, deviating significantly from the actual outputs.

**Figure 17A, B** displays the pitch angle estimation results. The UKF and ACKF algorithms exhibit poor estimation performance, whereas the LSTM-IPSO-UKF algorithm clearly shows errors between the estimated and true values in ground experiments. In comparison, the proposed algorithm yields smaller errors and maintains stable convergence, indicating overall better performance. As shown in **Figure 17B**, the absolute error of the UKF algorithm gradually increases as the platform’s operational time accumulates. Between t=20~50s, the error of the ACKF algorithm becomes significant, leading to reduced accuracy.

As shown in **Figure 18A, B**, the four algorithms exhibit similar variation patterns; however, the proposed algorithm’s estimated heading angles remain highly consistent with the actual outputs, demonstrating significantly better accuracy than the baseline algorithms. As seen in **Figure 18B**, due to initial errors and time accumulation, the errors of the LSTM-IPSO-UKF, UKF, and ACKF algorithms gradually diverge, leading to considerable deviations from the actual outputs.

**Tables 5**, **6** present the MAE and RMSE comparison results for attitude estimation across the four algorithms.

**Table 5:** Comparison of MAE and RMSE for attitude estimation among various algorithms.

Evaluation metric(deg)	Parameter	LSTM-IPSO-UKF	ACKF	UKF	Proposed algorithm
MAE	*roll*	0.2074	0.4607	0.8743	0.0917
	*pitch*	0.2828	0.6194	1.2851	0.1352
	*yaw*	6.0676	60.4530	31.8136	3.1458
RMSE	*roll*	0.2563	0.5521	1.0842	0.1132
	*pitch*	0.3620	0.7871	1.5258	0.1704
	*yaw*	33.9472	116.3719	68.3114	14.5289

**Table 6:** Performance improvement magnitudes of algorithms.

Evaluation metric(deg)	Parameter	LSTM-IPSO-UKF	ACKF	UKF
MAE	*roll*	55.79%	80.10%	89.51%
	*pitch*	52.19%	78.17%	89.48%
	*yaw*	48.15%	94.79%	89.56%
RMSE	*roll*	55.83%	79.50%	90.11%
	*pitch*	52.92%	78.35%	88.83%
	*yaw*	57.20%	87.52%	78.73%

As shown in **Tables 5**, **6**, the proposed algorithm markedly outperforms LSTM-IPSO-UKF, ACKF, and UKF across all attitude angles in terms of MAE and RMSE. Specifically, the MAE values for the roll, pitch, and heading angles are 0.0917°, 0.1352°, and 3.1458°, respectively, representing decreases of 55.79%, 52.19%, and 48.15% compared to the LSTM-IPSO-UKF algorithm, 80.10%, 78.17%, and 94.79% compared to the ACKF algorithm, and 89.51%, 89.48%, and 89.56% compared to the UKF algorithm. Similarly, the proposed algorithm reduced the RMSE for the roll angle estimate by 55.83%, 79.50%, and 90.11%; for the pitch angle by 52.92%, 78.35%, and 88.83%; and for the heading angle by 57.20%, 87.52%, and 78.73%, respectively.

#### 5.3.2 Speed parameter estimation results

**Figures 19**-**21** display the estimated results and absolute-error comparisons for the transport platform’s three-axis velocity at the center of mass across four algorithms.

As shown in **Figure 19**, all algorithms display consistent trends. However, LSTM-IPSO-UKF, UKF, and ACKF exhibit significant initial errors that grow and diverge over time. In contrast, the proposed algorithm closely follows the true value of *v_x_*, keeping errors within a narrow range throughout, thus demonstrating better estimation accuracy.

As shown in **Figure 20**, the trends of the algorithms are generally similar. However, the UKF and ACKF exhibit large initial errors that accumulate over time, resulting in poor estimation accuracy. The LSTM-IPSO-UKF displays significant errors during the period t=5~15s, with a rising trend. In contrast, the proposed algorithm’s errors remain consistently within a small range, demonstrating improved estimation accuracy for *v_y_*.

As shown in **Figure 21A, B**, because the vertical velocity of the transport platform varies only slightly, the four algorithms yield similar estimates of *v_z_*. However, compared with the LSTM-IPSO-UKF, ACKF, and UKF algorithms, the proposed algorithm has the smallest initial error and a maximum vertical velocity error that is much lower than those of the other benchmark algorithms. As seen in **Figure 21B**, the maximum errors of the other benchmark algorithms are relatively large, making it hard for them to meet engineering requirements.

**Tables 7**, **8** present the comparison results for MAE and RMSE in speed estimation across the four algorithms.

**Table 7:** Comparison of MAE and RMSE for speed estimation among various algorithms.

Evaluation metric(m/s)	Parameter	LSTM-IPSO-UKF	ACKF	UKF	Proposed algorithm
MAE	*v_x_*	0.1550	0.2571	0.2313	0.0622
	*v_y_*	0.1245	0.1360	0.2447	0.0616
	*v_z_*	0.0380	0.0301	0.0267	0.0209
RMSE	*v_x_*	0.2121	0.3058	0.2874	0.0861
	*v_y_*	0.1800	0.2192	0.2832	0.0916
	*v_z_*	0.0451	0.0389	0.0326	0.0272

**Table 8:** Performance improvement magnitudes of algorithms.

Evaluation metric(m/s)	Parameter	LSTM-IPSO-UKF	ACKF	UKF
MAE	*v_x_*	59.86%	75.80%	73.10%
	*v_y_*	50.49%	54.66%	74.81%
	*v_z_*	45.05%	32.51%	21.78%
RMSE	*v_x_*	59.38%	71.83%	70.03%
	*v_y_*	49.09%	58.19%	67.64%
	*v_z_*	39.63%	30.07%	16.55%

As shown in **Tables 7**, **8**, the proposed algorithm has the lowest MAE and RMSE for velocity estimation across all axes. Compared to the LSTM-IPSO-UKF, ACKF, and UKF algorithms, it provides velocity estimates with lower MAE and RMSE values.

Among these, the four algorithms— the proposed, LSTM-IPSO-UKF, ACKF, and UKF— produced mean absolute errors for *v_x_* estimates of 0.0622 m/s, 0.1550 m/s, 0.2571 m/s, and 0.2313 m/s, respectively. The relative mean errors for *v_y_* estimates were 0.0616 m/s, 0.1245 m/s, 0.1360 m/s, and 0.2447 m/s, respectively. The relative mean absolute errors for *v_z_* estimates were 0.0209 m/s, 0.0380 m/s, 0.0310 m/s, and 0.0267 m/s, respectively. Compared to the other three algorithms, the proposed algorithm improved the MAE for *v_x_* estimation by 59.86%, 75.80%, and 73.10%, respectively; enhanced the MAE for *v_y_* estimation by 50.49%, 54.66%, and 74.81%, respectively; and increased the MAE for *v_z_* estimation by 45.05%, 32.51%, and 21.78%, respectively. Similarly, the proposed algorithm improved the RMSE for *v_x_* estimation by 59.38%, 71.83%, and 70.03%, respectively; enhanced the RMSE for *v_y_* estimation by 49.09%, 58.19%, and 67.64%, respectively; and increased the RMSE for *v_z_* estimation by 39.63%, 30.07%, and 16.55%, respectively.

In summary, the proposed algorithm greatly enhances the accuracy of estimating dynamic center-of-mass parameters for tracked agricultural transport platforms. Especially in nonlinear operating conditions within unstructured agricultural environments, the algorithm offers precise estimates of attitude and velocity parameters with minimal, stable errors, demonstrating strong engineering practicality.

## 6 Conclusions

To overcome the challenges in directly measuring dynamic center-of-mass parameters and mitigating error accumulation issues in Inertial Measurement Units for tracked agricultural transport platforms in unstructured agricultural environments, this study proposes a hybrid estimation algorithm that combines the Unscented Kalman Filter with CNN-BiLSTM-Attention and the Improved Particle Swarm Optimization algorithm. The effectiveness of this approach is validated through ground-based real-vehicle experiments.

Based on the simulation and experimental results, the following conclusions can be drawn:

1. This study develops a CNN-BiLSTM-Attention hybrid data-driven model to replace the state and observation models in the traditional UKF algorithm. Bidirectional LSTM networks are adopted to learn time-dependent features, while attention mechanisms enhance the weighting of key dynamic features, effectively mitigating accuracy degradation under complex operating conditions.
2. To correct systematic errors caused by model mismatch, this study introduces a constant-coefficient compensation vector and uses IPSO with crossover and mutation operations from genetic algorithms for global optimization, enhancing convergence accuracy and stability of nonlinear state estimation.
3. Real-vehicle experiments under circular driving conditions were conducted based on the proposed hybrid estimation algorithm. The results show that the proposed algorithm achieves significantly lower MAE and RMSE in both attitude and velocity estimation than the conventional UKF, ACKF, and LSTM-IPSO-UKF algorithms, with errors remaining stable over time and showing no evident divergence, meeting the accuracy requirements for autonomous operations in unstructured agricultural environments.

Nevertheless, the proposed algorithm exhibits certain limitations in extreme scenarios. Its estimation accuracy may deteriorate under ultra-heavy loads and intense vibration due to sharply changed vehicle dynamics and aggravated sensor noise. The algorithm is therefore suitable for general farmland environments with moderate operating loads. Future work will improve the model robustness toward extreme working conditions to enhance its environmental adaptability.

In summary, the hybrid estimation algorithm introduced in this study offers an effective solution for real-time accurate perception of dynamic center-of-mass parameters for tracked agricultural transport platforms, which is crucial for developing intelligent agricultural transport control technologies and ensuring driving stability in complex operating conditions.

In summary, the hybrid estimation algorithm introduced in this study offers an effective solution for real-time accurate perception of dynamic center-of-mass parameters for tracked agricultural transport platforms, which is crucial for developing intelligent agricultural transport control technologies and ensuring driving stability in complex operating conditions.

## Data Availability

The raw data supporting the conclusions of this article will be made available by the authors, without undue reservation.
